# Integrated Phylogenomics and Expression Profiling of the MED Gene Family in *Brassica napus* Uncover Their Roles in Plant Development and Stress Tolerance

**DOI:** 10.3390/plants15182877

**Published:** 2026-09-20

**Authors:** Zhixing Jin, Yuning Wu, Ruisen Wang, Huiqi Zhang, Yang Zhu, Xiangtan Yao, Shengguan Cai, Imran Haider Shamsi, Cheng Qin

**Affiliations:** 1 College of Life and Environmental Sciences, Hangzhou Normal University, Hangzhou 311121, China; 2023111010031@stu.hznu.edu.cn (Z.J.); 2023111010063@stu.hznu.edu.cn (Y.W.); 2 Institute of Economic Crop Sciences, Jiaxing Academy of Agricultural Sciences, Jiaxing 314016, China; wangruisen@hotmail.com (R.W.); yxt156@hotmail.com (X.Y.); 3 Institute of Crop Science, Zhejiang University, Hangzhou 310058, China; zhq2022@zju.edu.cn (H.Z.); zhuyang2020@zju.edu.cn (Y.Z.); caisg@zju.edu.cn (S.C.); drimran@zju.edu.cn (I.H.S.)

**Keywords:** *MED* (*Mediator*) gene, bioinformatics analysis, *Brassica napus*, abiotic stress

## Abstract

Rapeseed (*Brassica napus* L.) is a globally vital oilseed crop for edible oil, biofuel and animal feed production, yet its yield and quality are severely threatened by a narrow genetic foundation, scarce germplasm and recurrent abiotic stresses including drought and salinity. Mediator subunits have been proven to be critical for abiotic stress tolerance in model plants, but systematic research on the *Brassica napus Mediator* (*BnaMED*) gene family and its stress regulatory mechanism in *Brassica napus* remains largely lacking. In this study, we identified 189 BnaMED members phylogenetically clustered into five subfamilies, with extensive family expansion mainly driven by whole-genome duplications, accompanied by functional differentiation and redundancy to bolster polyploid adaptability. Chromosomal mapping revealed 176 *BnaMED* genes unevenly distributed across A/C subgenomes. Through gene collinearity analysis among *Brassica napus*, *Arabidopsis thaliana*, *Glycine max*, *Oryza sativa*, and *Zea mays*, we found that *Brassica napus* shares high collinearity with dicotyledonous species, such as *Glycine max* and *Arabidopsis thaliana*, indicating evolutionary conservation specific to dicots. Protein physicochemical properties and promoter cis-element analysis uncovered diverse structural features and extensive involvement in light, hormone and stress signaling pathways. Tissue expression profiling demonstrated distinct subfamily specificity and functional divergence. The quantitative real-time PCR (qRT-PCR) analysis revealed that under salt stress, endogenous transcript levels of BnaMED family members *BnaA09g34000D*, *BnaA09g23620D*, *BnaC03g31860D* and *BnaA06g10800D* were up-regulated, whereas *BnaA08g23170D* and *BnaA08g22420D* were transcriptionally repressed. Following drought-stress treatment, all six genes (*BnaA09g34000D*, *BnaA09g23620D*, *BnaA09g50540D*, *BnaA06g10800D*, *BnaA08g23170D* and *BnaA08g22420D*) displayed markedly reduced expression. These observations reflect stress-specific regulatory strategies underlying abiotic-stress adaptation. In addition, several *BnaMED* genes showed dynamic expression during floral transition in the semi-winter Zhong Shuang 11 (ZS11) cultivar. This study provides a foundational insight into functional roles of *BnaMED* genes in rapeseed abiotic stress responses.

## 1. Introduction

Rapeseed is a crucial oil-bearing crop cultivated globally, accounting for 13% of global oil production, second only to soybean (28%) [1]. It is cultivated across approximately 35 million hectares in regions including China, Europe, Canada and Australia, with an impressive annual output of up to 70 million tons of oilseeds. *Brassica napus* (oilseed rape, *A_n_A_n_C_n_C_n_*, 2n = 4x = 38) is regarded as one of the most vital oilseed crops globally, owing to its advantages including disease resistance, stress tolerance and broad applicability. It is widely utilized as a pivotal crop for the production of edible oil, vegetable products, biofuels and animal feed [2]. *B. napus* originated from an interspecific hybridization event between two diploid progenitors, *Brassica rapa* (*A_r_A*_r_, 2n = 2x = 20) and *Brassica oleracea* (*C_o_C_o_*, 2n = 2x = 18), followed by spontaneous chromosome doubling, which led to the formation of this novel amphidiploid species [3,4]. However, rapeseed production in China is confronted with critical challenges, including a narrow genetic basis, lack of elite germplasm resources, and restricted growing windows [5]. Thus, *B. napus* exhibits extremely high research value in both biological and agricultural sciences, and more systematic and in-depth research and exploration are urgently required.

Eukaryotes are subject to the influence of either endogenous or exogenous factors, which induce substantial alterations in gene expression levels. Such changes are predominantly regulated by DNA-binding transcription factors (TFs). Furthermore, the multiprotein Mediator complex, acting as a signal integrator, exerts transcriptional regulatory effects by transmitting signals from TFs to RNA polymerase II (Pol II) [6]. Kornberg and colleagues first identified Mediator in yeast in the 1990s and revealed that this complex is capable of relieving competition between activators for a common target [7]. Accumulating research evidence has demonstrated that Mediator is widely distributed in mammals and a variety of plant species. Structural studies indicated that Mediator subunits form stable subcomplexes comprising three main modules (Head, Middle, and Tail). The modular organization of Mediator may reflect the different functions of Mediator components. The Head module stabilizes the conformation of the transcriptional initiation complex through binding to Pol II; the Middle module extends to the Pol II foot and may influence polymerase conformation; and the Tail module is considered to be targeted by specific TFs [8,9,10,11,12,13]. The Mediator structure can shift dramatically on binding to other proteins or protein complexes. These structural rearrangements are essential for Pol II holoenzyme formation and the capacity of Mediator to integrate multiple regulatory signals [14,15].

The first plant Mediator complex was isolated from *Arabidopsis thaliana* in 2007. Björklund and colleagues performed biochemical purification of the complex and identified 21 conserved and six plant-specific Mediator subunits in *A. thaliana* [16]. In subsequent studies, multiple research groups have identified 8 subunits homologous to Mediator proteins of yeast and metazoans, including MED12, MED13, Cyclin-dependent kinase 8 (CDK8), CycC, MED26, MED30, MED32, and MED33. Therefore, the *A. thaliana* Mediator complex currently comprises 29 conserved and four plant-specific subunits [10,17]. Comparative genomics analyses indicate that homologs of the *A. thaliana* Mediator subunits are expressed in diverse plant species, such as *Oryza sativa* L. Furthermore, the subunit sequences displayed high similarity across different plants, suggesting that the function of the corresponding subunit is conserved in various plants [18]. With the advancement of in-depth research on plant Mediator, it has been proved that they participate in multiple biological processes including flowering, defense, abiotic stress, genomic stability, metabolism homeostasis, growth and development [18,19,20,21,22]. For example, the *MED14* gene is involved in the formation of meristem patterning and in controlling the duration of cell proliferation. The *med14* mutant showed a dwarf phenotype and abnormal shoot development due to alterations in the cell number [23]. The *MED25* gene integrates various regulatory processes, such as flowering, hormone signaling pathways, and biotic/abiotic stress responses. Specifically, it promotes flowering in *A. thaliana* in response to light signals. A separate role for MED25 in pathogen defense has been demonstrated; *med25* mutants exhibit enhanced susceptibility to the foliar necrotrophic fungal pathogens *Alternaria brassicicola* and *Botrytis cinerea*, while displaying increased resistance to the root-infecting hemibiotrophic fungal pathogen, *Fusarium oxysporum* [24,25]. In addition, *MED25* regulates the expression of multiple class III peroxidase and NADPH oxidase genes, as well as reactive oxygen species (ROS) distribution in vivo, which is crucial for root hair development, consequently modulating nutrient uptake and overall plant growth [26]. Additionally, several Mediator subunits, including *MED8*, *MED17*, and *MED18*, play critical roles in flowering regulation. Among them, *MED8* and *MED21* also function in biotic stress responses, whereas *MED16* is implicated in abiotic stress responses [25,27,28].

As global warming intensifies and extreme events such as droughts and floods become more frequent, crops face growing environmental uncertainties throughout their growth cycle. This has caused massive crop losses during cultivation and drastically restricted the regular progress of agricultural production. Therefore, generating mutant plants with enhanced tolerance to harsh environments has become a crucial current objective. Previous studies have highlighted the importance of maintaining proper water balance for improving productivity in *B. napus*. However, drought stress reduces seed germination rate, shoot length, and root length, while enhancing electrolyte leakage, respiration rate and reactive oxygen species (ROS) accumulation. Ultimately, these changes disrupt physiological processes in *B. napus* and severely inhibit its growth and development [29,30,31]. In addition to drought, salt stress is also recognized as one of the major abiotic stresses restricting plant growth and productivity, especially in arid and semi-arid regions [32,33]. Salt stress mainly restricts photosynthesis and CO_2_ diffusion by reducing stomatal conductance and the mesophyll conductance. It also causes oxidative damage through the production of ROS. Stressed plants synthesize antioxidants in cells to scavenge ROS and resist such stress [34,35]. According to related reports, members of the MED family play a key role in the response to abiotic stress. The *med25* mutant exhibits a prominent drought-resistant phenotype in both *A. thaliana* and *O. sativa*. Specifically, under drought stress, the *med25* mutant can up-regulate the expression of drought-responsive genes including *RD29A*, *RD29B*, and *DREB2A*. Moreover, *MED25* exerts a significant positive regulatory effect on salt stress resistance. It interacts with TFs such as DREB2A, ZINC FINGER HOMEODOMAIN 1 (ZFHD1), and MYB-like proteins, thereby promoting the strong expression of salt stress-responsive genes [36]. Additionally, CDK8 positively regulates the drought stress response in *A. thaliana* by interacting with either the RAP2.6-SnRK2.6 complex or WIN1 [37,38]. MED2, MED14, and MED16 function as essential Mediator subunits required for ABRE-dependent gene activation. Their simultaneous mutation confers an ABA-insensitive phenotype in the triple mutant, which displays a markedly increased water loss rate in detached leaves relative to the wild type [39]. The *A. thaliana med18* mutant displays sensitivity to salt stress and ABA. Recent studies have shown that, relative to wild-type plants, numerous Mediator complex subunit mutants display substantial alterations in the enrichment levels of several transcription factor binding sites during the early phase of the salt stress response. More specifically, *MED18* plays a key role in this process, and it is essential for the transcriptional regulation mediated by abiotic stress-associated transcription factor families, including bZIP, bHLH, and MYB [40]. To date, studies investigating the roles of Mediator subunits in abiotic stress responses have been largely restricted to *A. thaliana* and *O. sativa*, with relatively few reports elucidating the functional mechanisms of the *BnaMED* family in *B. napus* under abiotic stress.

In this study, we systematically identified 189 *BnaMED* genes exhibiting high sequence homology with their *A. thaliana* counterparts in the *B. napus* cv. Westar genome using bioinformatic approaches. Comprehensive analyses were performed to characterize their gene structures and chromosomal distributions, and a multispecies phylogenetic tree was constructed across four representative species, including *A. thaliana*, *G. max*, *O. sativa*, and *Z. mays*. Furthermore, quantitative expression profiling revealed stress-specific expression patterns across the *BnaMED* family, with several key genes exhibiting distinct responses indicative of functional diversification under multiple abiotic stresses. Our findings not only fill a critical knowledge gap in the study of the *MED* gene family in rapeseed, but also lay a solid foundation for exploring potential functions of *MED* subunits in stress tolerance mechanisms in *B. napus*.

## 2. Results

### 2.1. Identification and Subfamily Classification of 189 Mediator Family Members in B. napus (cv. Westar)

To identify the Mediator complex (MED) family members in *B. napus*, the amino acid sequences of AtMED proteins were used as queries to perform a BLAST search against the *B. napus* genome database, with an E-value less than 1.0 × 10^−5^. Conserved domains and motifs were analyzed using the NCBI CDD database and TBtools-II (v2.382). Subunits containing the conserved domains were designated as members of the *MED* gene family. Thus, 189 MED family members in *B. napus* were identified.

**Figure 1:**
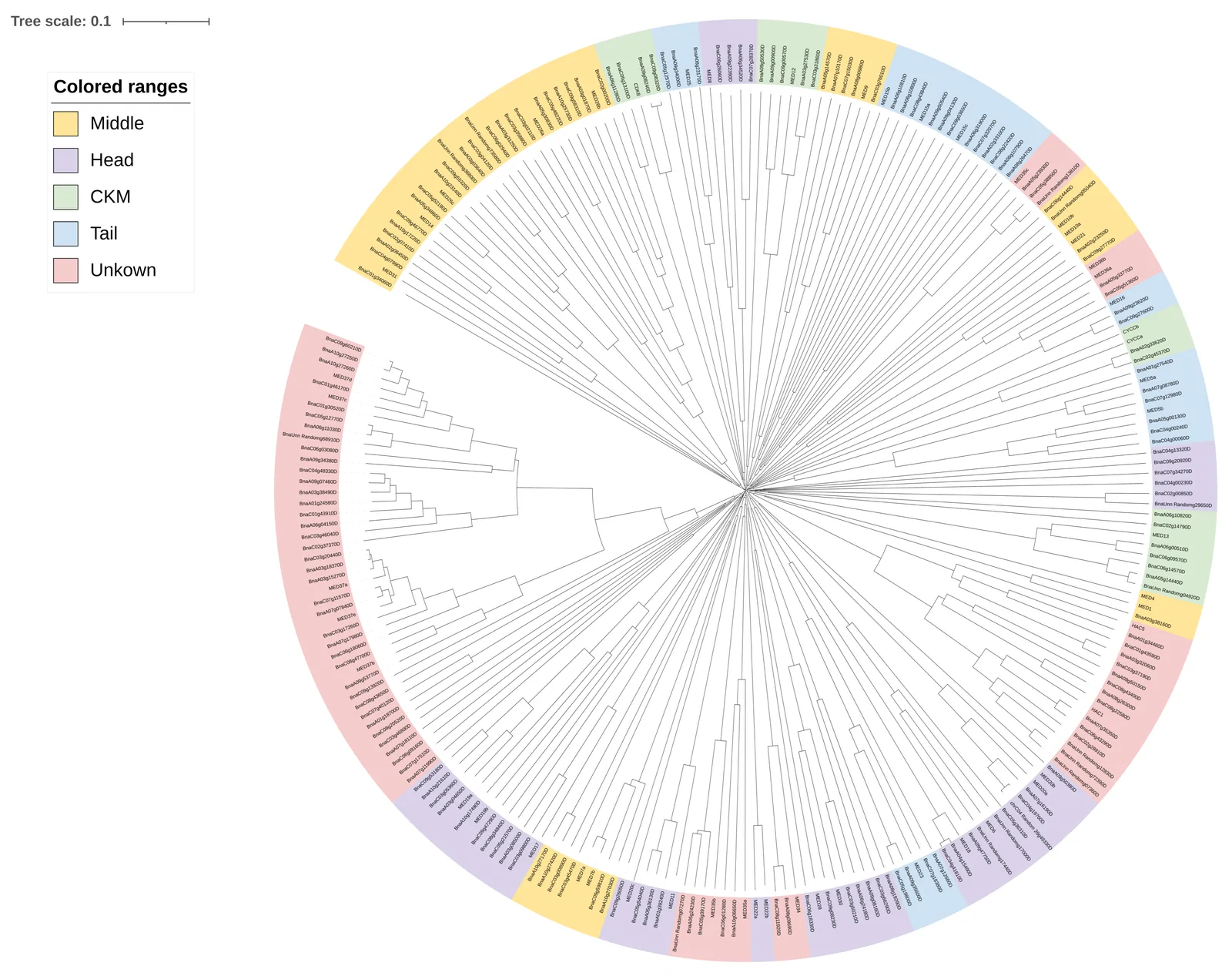
Phylogenetic analysis of the *MED* gene family between *B. napus* and *A. thaliana*. Multiple protein sequence alignment and phylogenetic tree construction were conducted using MEGAX64 software with the neighbor-joining algorithm and 1000 bootstrap replicates. Different colors represent the five subfamilies of the *MED* gene family.

To further clarify the evolutionary relationships among MED family members in *B. napus*, we performed multiple sequence alignments of MED proteins from *B. napus* and *A. thaliana* and constructed an unrooted phylogenetic tree using MEGA X64 software. Based on the modular classification of the *A. thaliana* Mediator complex, the BnaMED family was divided into five subfamilies, namely Head, Middle, Tail, CDK8 kinase module (CKM), and Unknown. Phylogenetic analysis showed the distribution of members across each subfamily as follows. The Head subfamily contained 49 members, 35 of which were from *B. napus*; the Middle subfamily included 58 members, with 44 from *B. napus*; the Tail subfamily comprised 39 members, among which 31 were *B. napus* proteins; the CKM subfamily possessed 18 members, 13 of which belonged to *B. napus*; and the Unknown subfamily contained 79 members, with 66 derived from *B. napus* (Figure 1).

The results of this study revealed remarkable expansion of the *MED* gene family in *B. napus*, as evidenced by the substantial number of its 189 members relative to its diploid progenitor *A. thaliana*. This extensive expansion of the gene family was likely driven by multiple rounds of whole-genome duplication events during the evolutionary divergence, speciation, and domestication of *B. napus*. Previous studies have validated that homologous genes derived from such duplication events are not merely functionally redundant. A subset tend to drive the specialization of specific biological functions through the divergence of sequences and expression patterns, while others maintain functional overlap and construct a genetic redundancy network to ensure the stability of key agronomic traits.

### 2.2. Gene Structure of the BnaMEDs

Notably, the AtMED family is classified as a functionally clustered gene group rather than a sequence-homologous family, which accounts for the low overall sequence conservation among its members. Therefore, it is challenging to deduce the inherent characteristics of the orthologous MED family in *B. napus* via conserved motif analysis. To resolve this issue, we shifted our research focus to gene structure analysis, aiming to reveal the potential correlations between gene structural features and their functional attributes.

**Figure 2:**
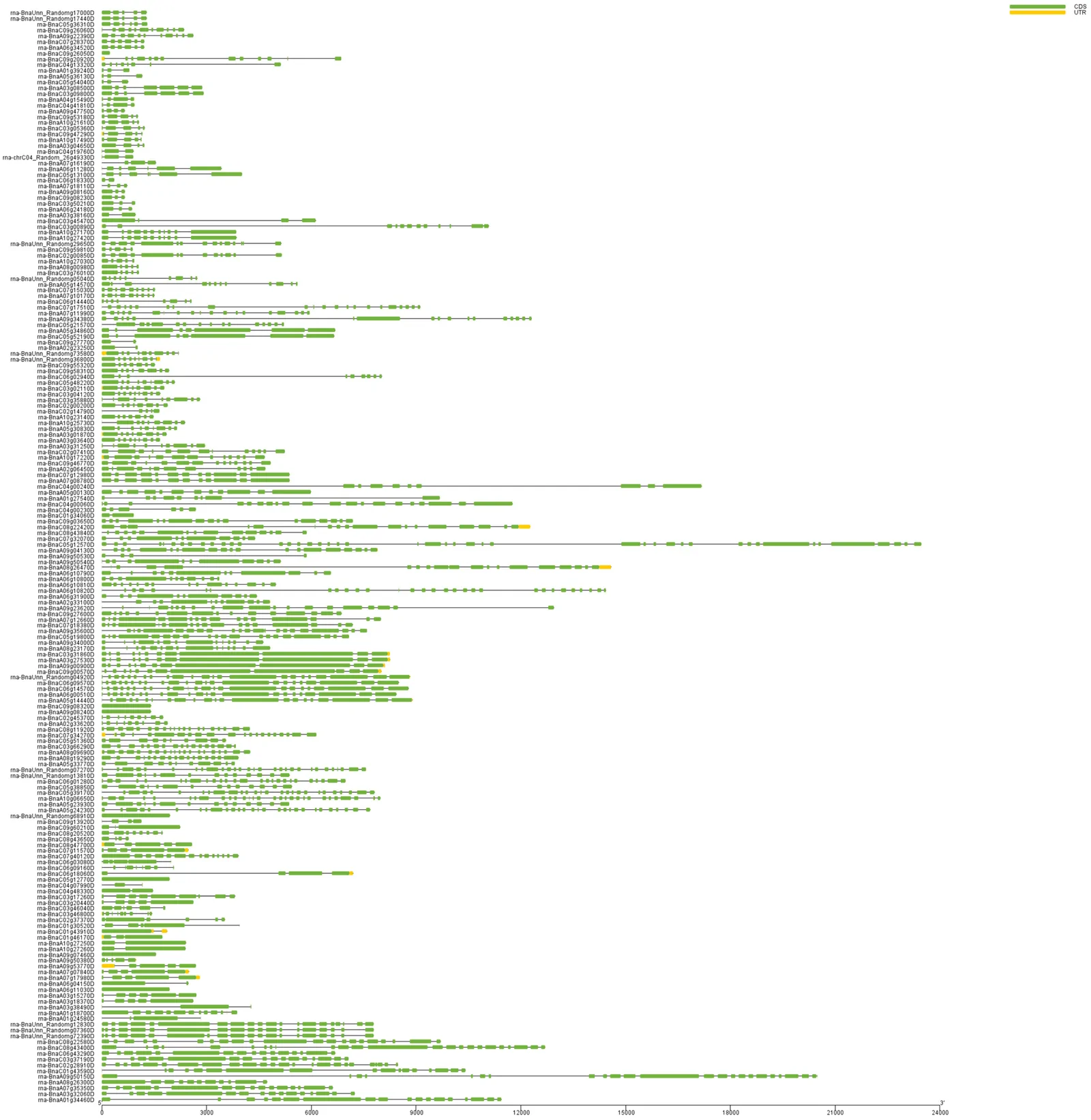
Visualization of gene structures in the BnaMED family. Green boxes denote exons, gray lines denote introns, and yellow boxes at both termini represent 5′-UTR and 3′-UTR regions, respectively. The scale bar indicates the length of the gene sequences.

Visualization of gene structures across the BnaMED family was conducted using TBtools-II v2.382 software. The results demonstrated considerable structural diversity among these genes. The vast majority of BnaMED members contained three or more exons, consistent with the canonical structural features of eukaryotic genes (Figure 2). Nevertheless, we also identified a small subset of single-exon genes (e.g., *BnaC09g08320D* and *BnaA09g08240D*), representing a minor deviation from the typical exon-intron organization of eukaryotic genes.

In summary, the BnaMED family exhibited obvious heterogeneity in gene structure. The predominantly multi-exon structure provides a structural foundation for alternative splicing, which may contribute to functional diversification of the family within complex regulatory networks. In contrast, the small number of single-exon genes display more compact structures, suggesting distinctly different regulatory mechanisms underlying their expression.

### 2.3. Chromosome Distributions of the BnaMED Genes

**Figure 3:**
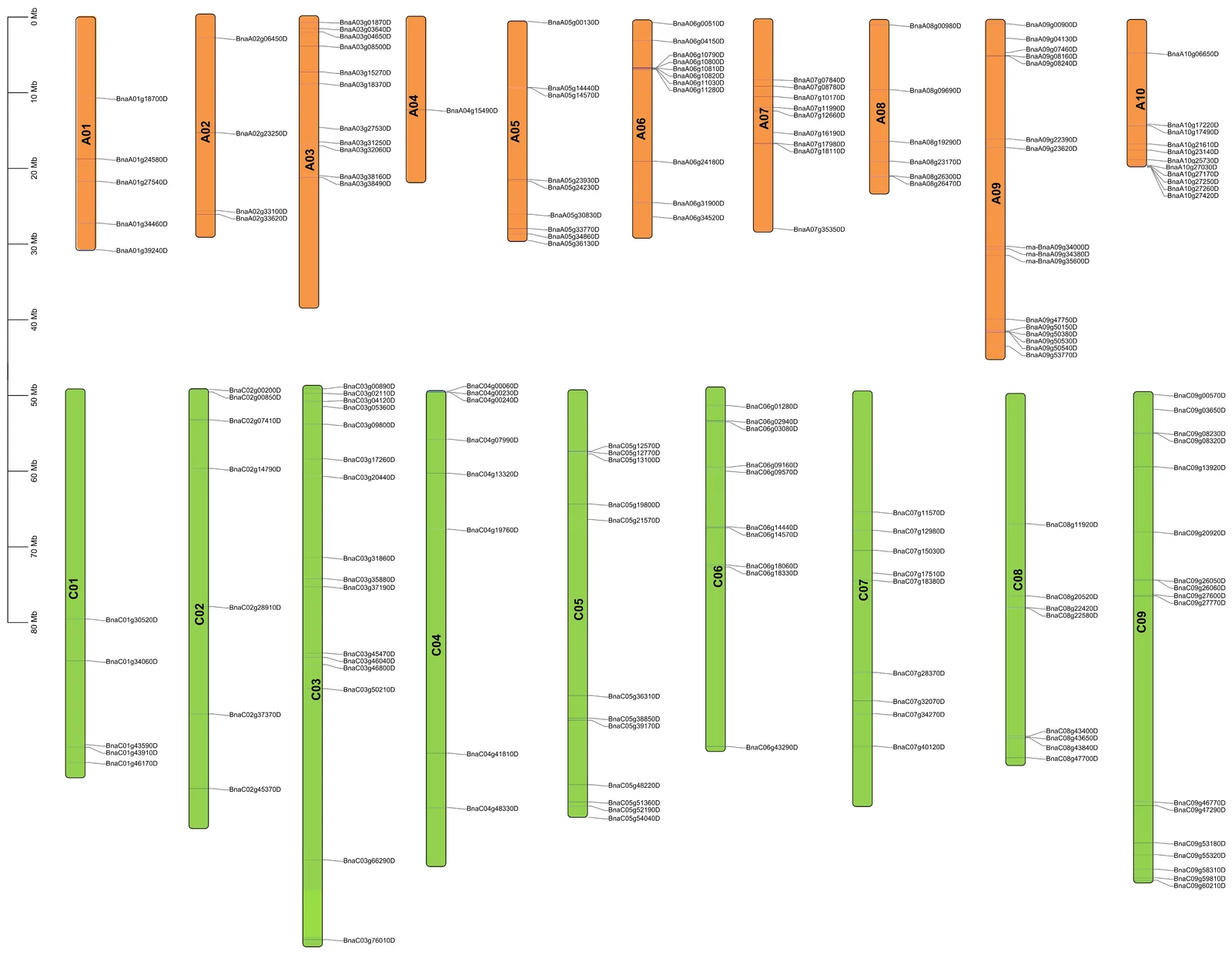
Chromosome locations of *BnaMED* family genes. The bar length represents the size of each chromosomes in *B. napus*. Physical positions of genes are shown on the left side of each chromosome, with corresponding gene names labeled on the right.

To intuitively characterize the chromosomal distribution of *BnaMED* genes in *B. napus*, a chromosomal distribution map was generated using TBtools-II v2.382 of the 189 *BnaMED* genes, 176 were mapped to either the A or C subgenome (83 in the A subgenome and 93 in the C subgenome), with the remaining 13 genes being unanchored. These genes exhibited an uneven distribution across chromosomes; the highest gene density was observed on chromosome C09 (17 genes), whereas only one gene was detected on chromosome A04 (Figure 3).

Further analysis revealed that the distribution pattern of *BnaMED* genes is likely closely associated with their evolutionary history. A considerable number of collinear homologous gene pairs were identified between the A and C subgenomes, which directly confirms that the expansion of this gene family was primarily driven by whole-genome duplication events during the formation of *B. napus*.

### 2.4. Synteny Analysis of BnaMED Genes

**Figure 4:**
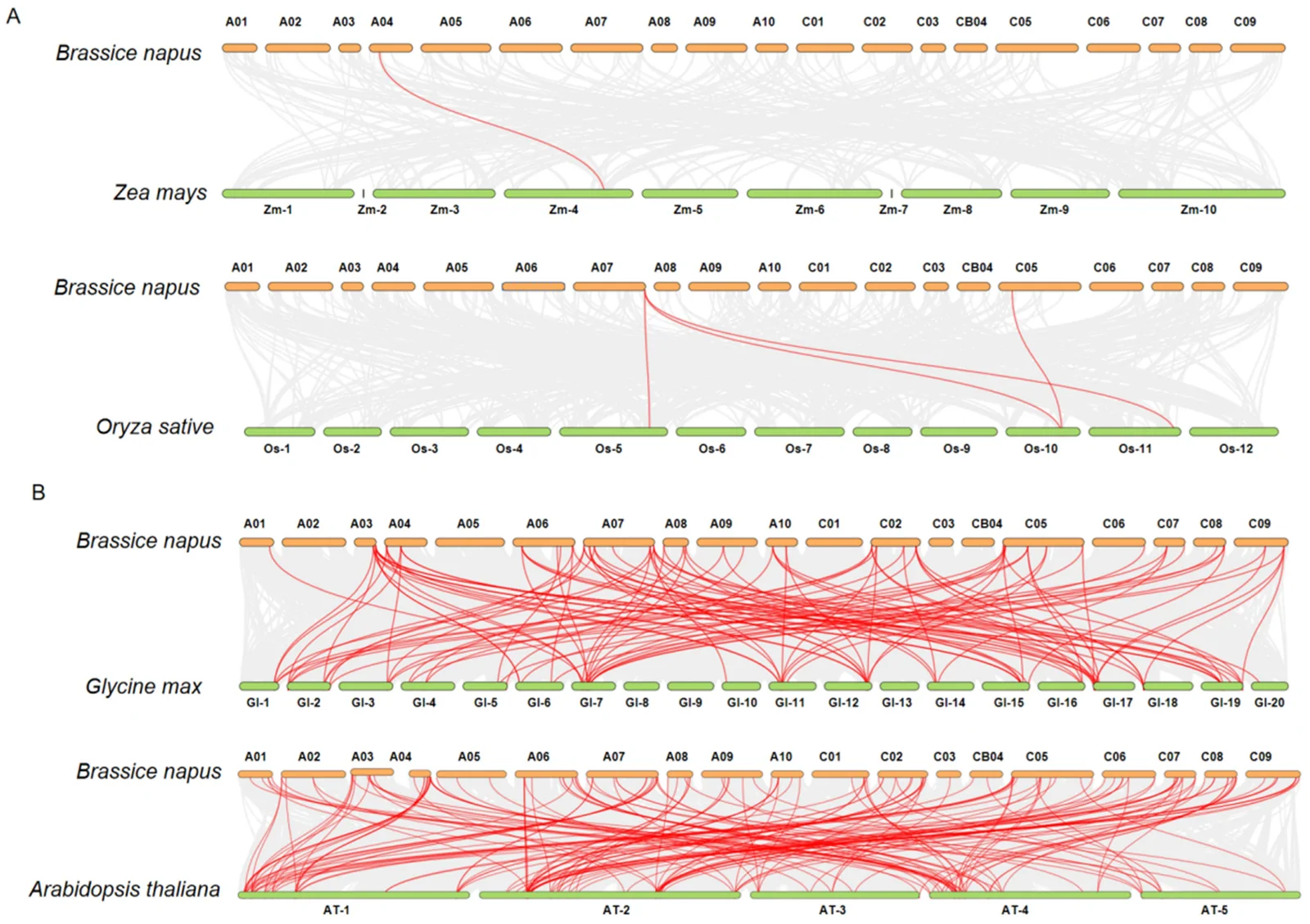
Analysis of syntenic relationships among *O*. *sativa*, *Z*. *mays* L. (**A**), *A. thaliana*, and *G. max* (**B**). Gray lines represent syntenic blocks across plant genomes, while red lines represents collinear *MED* gene pairs.

To further investigate the phylogenetic relationships of the *B. napus* MED family, we constructed a collinearity map between *B. napus* and four representative species, including two monocots (*O. sativa* L. and *Z. mays*) and two dicots (*A. thaliana* and *G. max*). The results revealed substantial variation in the number of conserved collinear homologous gene pairs between *B. napus* and the examined species. Only one pair was identified between *B. napus* and *Z. mays*, and four pairs between *B. napus* and *O. sativa.* By contrast, collinear gene pairs between *B. napus* and the two dicotyledonous species (*A. thaliana* and *G. max*) were far more numerous than those detected between *B. napus* and two monocotyledonous species (Figure 4).

This pattern clearly indicates that the number of orthologous genes shared among dicotyledonous plants is far greater than that between dicots and monocots. This is consistent with the evolutionary principle that closer phylogenetic relationships correspond to higher levels of collinearity. In addition, most collinear gene pairs identified between *B. napus* and *A. thaliana* were also extensively conserved between *B. napus* and *G. max*. This further supports that these genes originated in the common ancestor of dicotyledonous plants and have remained relatively conserved during subsequent species diversification. Therefore, the evolutionary history of the *B. napus* MED family is closely associated with the diversification of dicotyledonous lineages, and its pattern of gene retention and loss faithfully reflect the phylogenetic relationships among these species.

### 2.5. The Biophysical Properties of BnaMEDs

Using TBtools-II v2.382, we analyzed the biophysical properties of 189 BnaMED family members systematically. The molecular weights of BnaMED proteins exhibited an extremely wide distribution, ranging from 8.52 kDa (*BnaC09g26050D*) to 311.67 kDa (*BnaC05g12570D*), implying that this family may include both small regulatory proteins participating in rapid signal transduction and large structural proteins serving as core components of protein complexes. The isoelectric points (pI) ranged from 4.48 (*BnaA06g24180D*) to 9.84 (*BnaA06g10800D*). According to the pI values, the number of acidic proteins (107) was higher than that of basic proteins (81), indicating that most BnaMED proteins are acidic. Instability index analysis showed that most proteins exhibited an instability index greater than 40, implying that these proteins may have relatively short intracellular half-lives. Their functions are therefore likely dynamically regulated at the transcriptional or post-translational levels. Most proteins showed negative GRAVY values, indicating that BnaMED proteins are predominantly hydrophilic. The aliphatic index varied over a broad range (46.79–124.32), reflecting considerable divergence in thermal stability and structural rigidity among family members. This may underpin their functional adaptation to diverse subcellular localizations and temperature regimes (Table 1).

### 2.6. Cis-Regulatory Elements Identification in Promoter

To clarify the transcriptional regulatory basis of the *BnaMED* gene family, we analyzed the cis-acting elements harbored in their promoter regions. Specifically, we extracted the 2000 bp sequences upstream of the translation initiation codon (ATG) for all *BnaMED* genes. Subsequently, we identified and annotated the cis-regulatory elements in these regions using TBtools-II v2.382 combined with the PlantCARE database.

**Figure 5:**
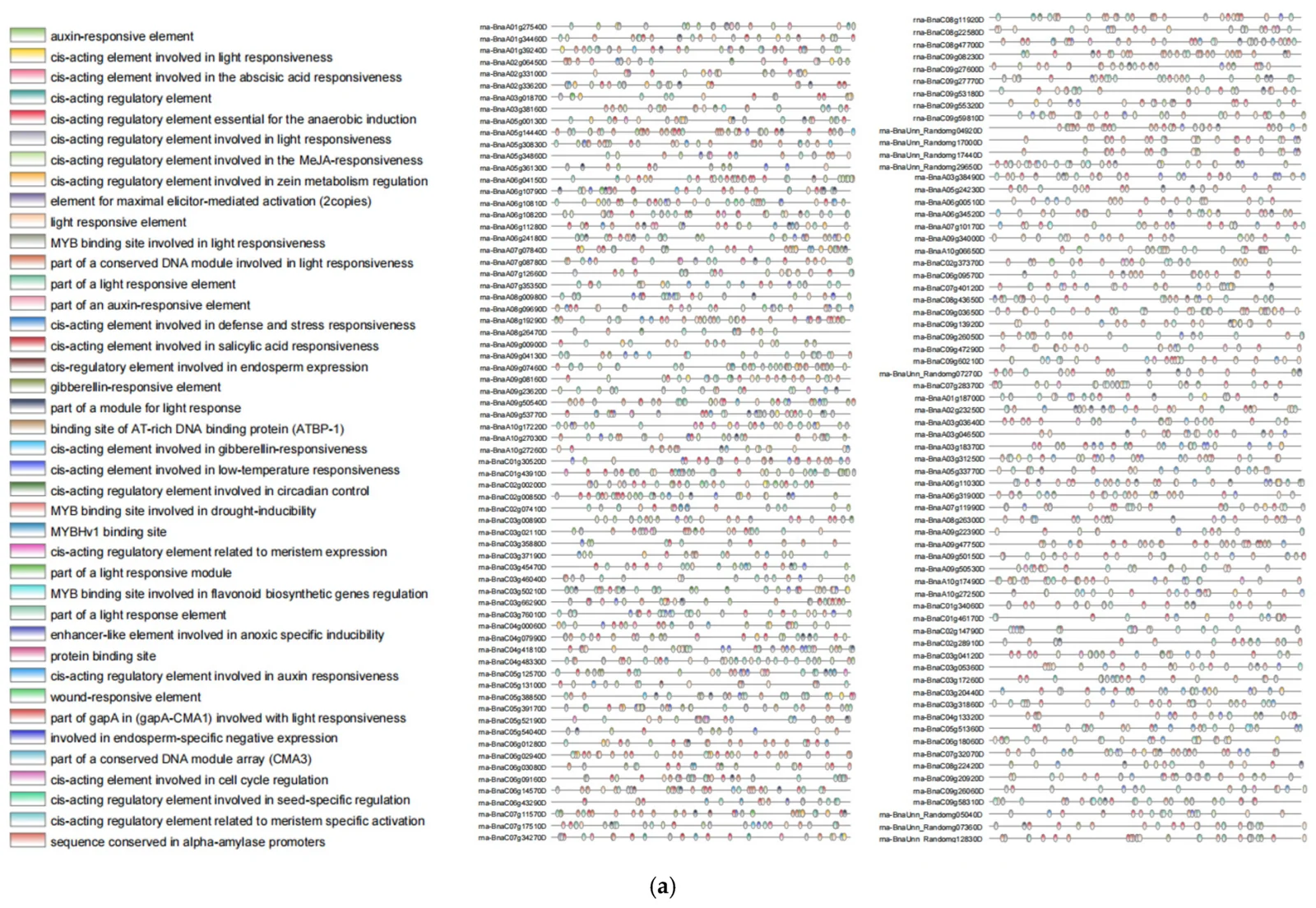

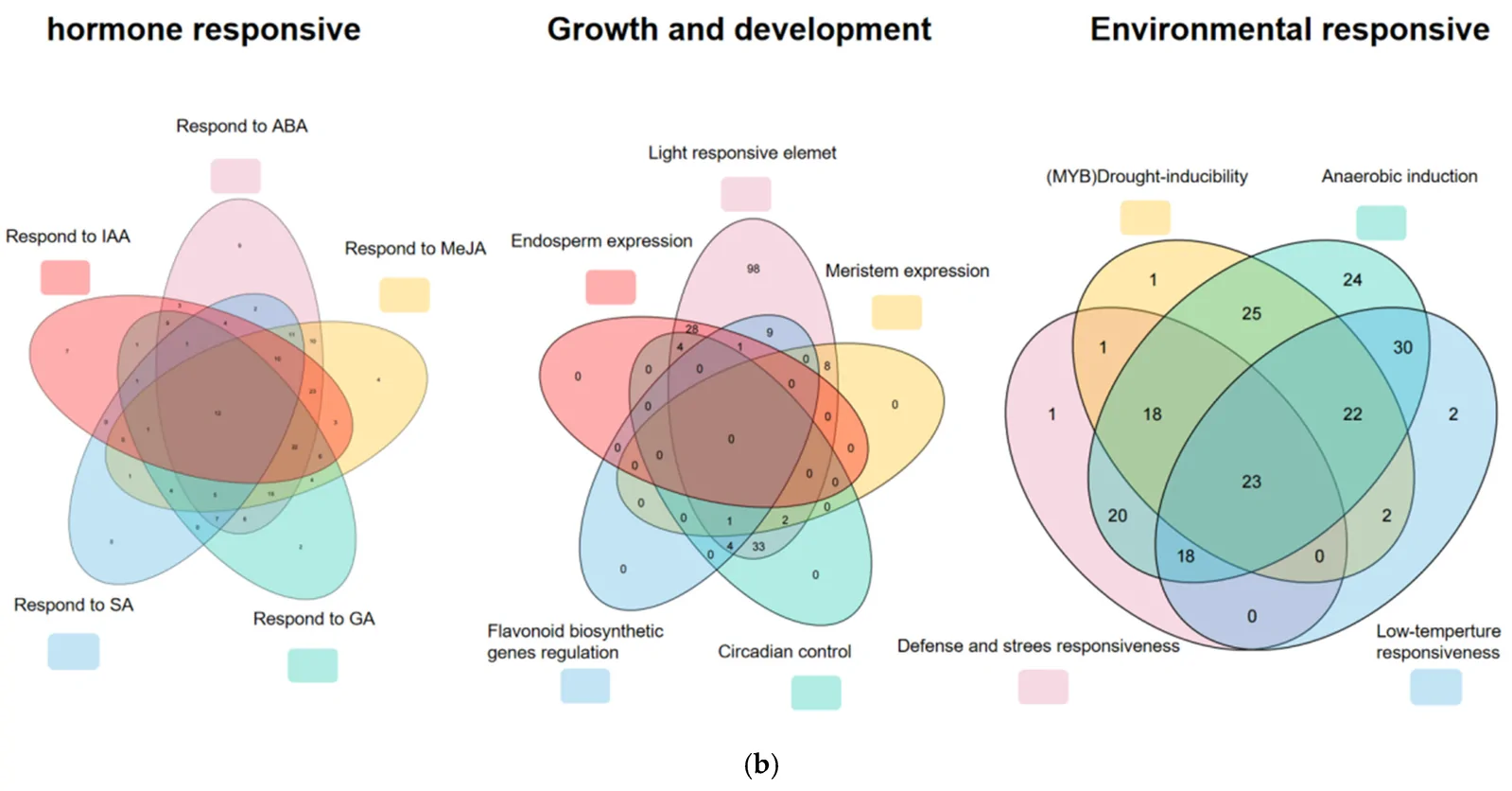
Cis-elements analysis in the promoter regions of *BnaMED* genes. (**a**) Visualization of cis-acting elements in the *BnaMED* gene family using TBtools-II v2.382. (**b**) Venn diagram showing the distribution of cis-acting elements associated with different biological pathways.

As predicted, the promoters of all *BnaMED* genes contained core conserved promoter elements, such as TATA-box and CAAT-box. Among the total 7732 cis-acting elements identified, light-responsive elements displayed the highest abundance and diversity, including 3-AF1 binding site, AE-box, Box II, and other typical motifs. These elements were detected in nearly all *BnaMED* genes. This result strongly suggests that the BnaMED family is extensively involved in the light-signaling regulatory network of *B. napus*. Furthermore, a large number of hormone-responsive elements were identified, including motifs associated with methyl jasmonate (MeJA, 530 elements), abscisic acid (ABA, 400 elements), gibberellin (GA, 117 elements), auxin (IAA, 116 elements) and salicylic acid (SA, 89 elements). These results indicate that the BnaMED family plays important regulatory roles in multiple phytohormone signaling pathways. Meanwhile, diverse stress-responsive elements were also distributed in the promoter regions, including TC-rich repeats related to defense and stress responses, MBS motifs associated with drought inducibility, ARE elements involved in anaerobic induction, and LTR elements responsive to low temperature. Collectively, these findings illustrated that the *BnaMED* gene family likely contributes to the adaptation of *B. napus* to various abiotic stresses (Figure 5, Table S2).

Taken together, the promoters of *BnaMED* genes exhibit a highly modular organization of cis-acting elements, with substantial enrichment of light-responsive, hormone-responsive, and stress-responsive elements. Our results support the critical role of the BnaMED family in integrating light signals, hormonal pathways, and stress responses at the transcriptional regulatory level, which may underlie its functional diversity in the adaptation of polyploid *B. napus* to complex environments. These findings provide valuable insights for the future identification of key regulatory genes and the elucidation of their transcriptional regulatory mechanisms under diverse conditions.

### 2.7. Expression Profiling Analysis of BnaMED Genes Across 12 Distinct Tissues

Gene expression patterns are critical for elucidating gene functions. To explore the potential roles of the *BnaMED* gene family in *B. napus* cv. Westar, family members were identified by homologous alignment using the public transcriptome database of *B. napus* cv. Zhongshuang 11 (ZS11). We used the screened homologous genes to compare with the ZS11 database to find the sequence with the highest homology to our sequence, and then found its information in the database to make a heat map. Their expression profiles across 12 tissues (including roots, stems, leaves, floral organs and seeds) were subsequently analyzed using TBtools-II v2.382.

**Figure 6:**
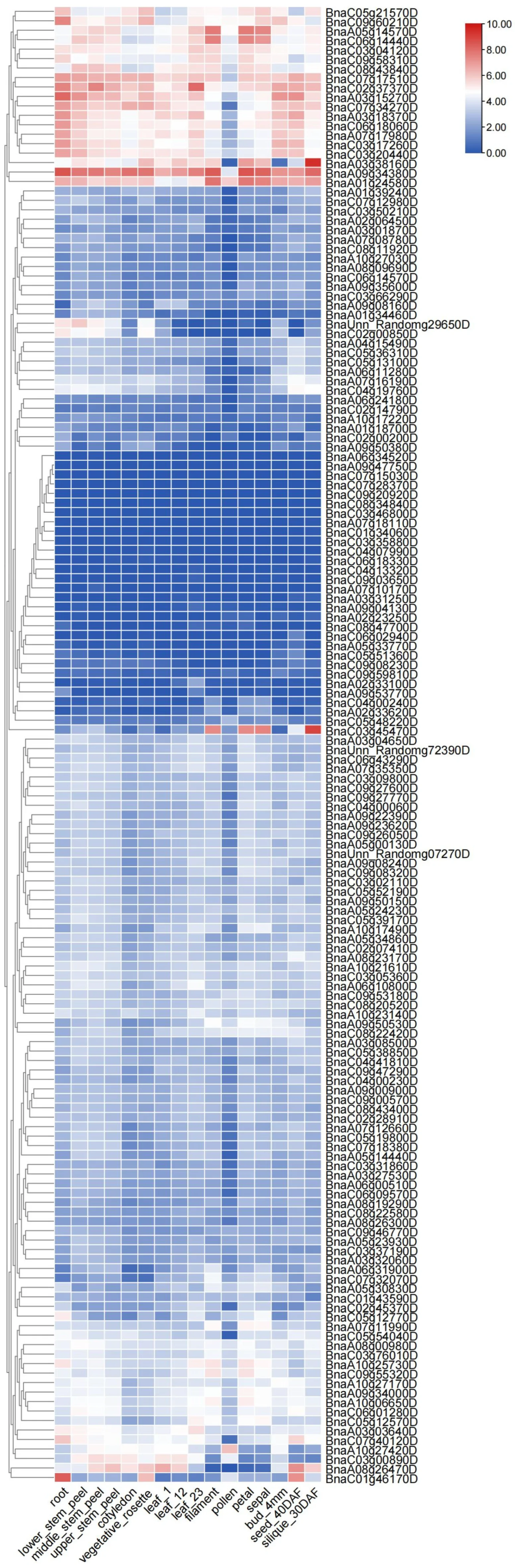
Expression patterns of *MED* family genes in 12 tissues. The heat map was constructed based on log_2_ (TPM + 1) expression values. The color bar represents the log_2_ (TPM + 1) expression level, with the legend shown on the right side of the figure. Genes with high expression levels are labeled in red, while those with low expression levels are labeled in blue. Family members are identified in *B. napus* cv. Zhongshuang 11 (ZS11) by homologous alignment using public transcriptome database.

Heat map analysis revealed that the expression patterns of *BnaMED* genes displayed distinct tissue specificity and an obvious trend of functional divergence. Several genes, including *BnaC03g17260D*, *BnaC06g18060D*, *BnaC09g60210D*, *BnaA03g15270D*, and *BnaA03g38160D*, exhibited constitutively high expression across most tested tissues. These genes mainly belonged to the Unknown subfamily, with only a small proportion belonging to the Middle subfamily, suggesting that they may be involved in fundamental physiological processes in *B. napus*. In contrast, several other genes (e.g., *BnaC07g28370D*, *BnaA06g34520D*, *BnaC08g34840D*, *BnaC08g47700D*) exhibited overall low expression levels in all examined tissues. These genes were distributed in the Head and Middle subfamilies, with only a small number classified into the Tail and Unknown subfamilies (Figure 6). Notably, members of the Head and Middle modules, which perform essential functions in *A. thaliana*, exhibited relatively weak expression in *B. napus*, which may reflect functional redundancy and divergence within transcriptional regulatory networks following polyploidization. The vast majority of *BnaMED* genes showed low expression levels in pollen, indicating that this gene family may not be involved in pollen development but instead participates more broadly in transcriptional regulation in other tissues.

In summary, the *BnaMED* gene family exhibits extensive heterogeneity in expression patterns and functional module reorganization in *B. napus*. Some *BnaMED* genes retain conserved basal functions through constitutive expression, while others appear to have evolved into potential functional reservoirs or novel functional units via expression repression or neofunctionalization during polyploidization. These results further support the role of whole-genome duplication in driving gene expression divergence and adaptive evolution, and provide a critical candidate gene resource for future functional studies aimed at specific tissues or stress conditions.

### 2.8. Transcriptional Response of MED Gene to Abiotic Stress in B. napus

To investigate the transcriptional responses of *BnaMED* genes to drought and salt stress, several homologous genes harboring typical stress-responsive cis-elements and previously reported to be involved in stress tolerance were selected. Their expression patterns at multiple time points under stress treatments were analyzed using qRT-PCR.

**Figure 7:**
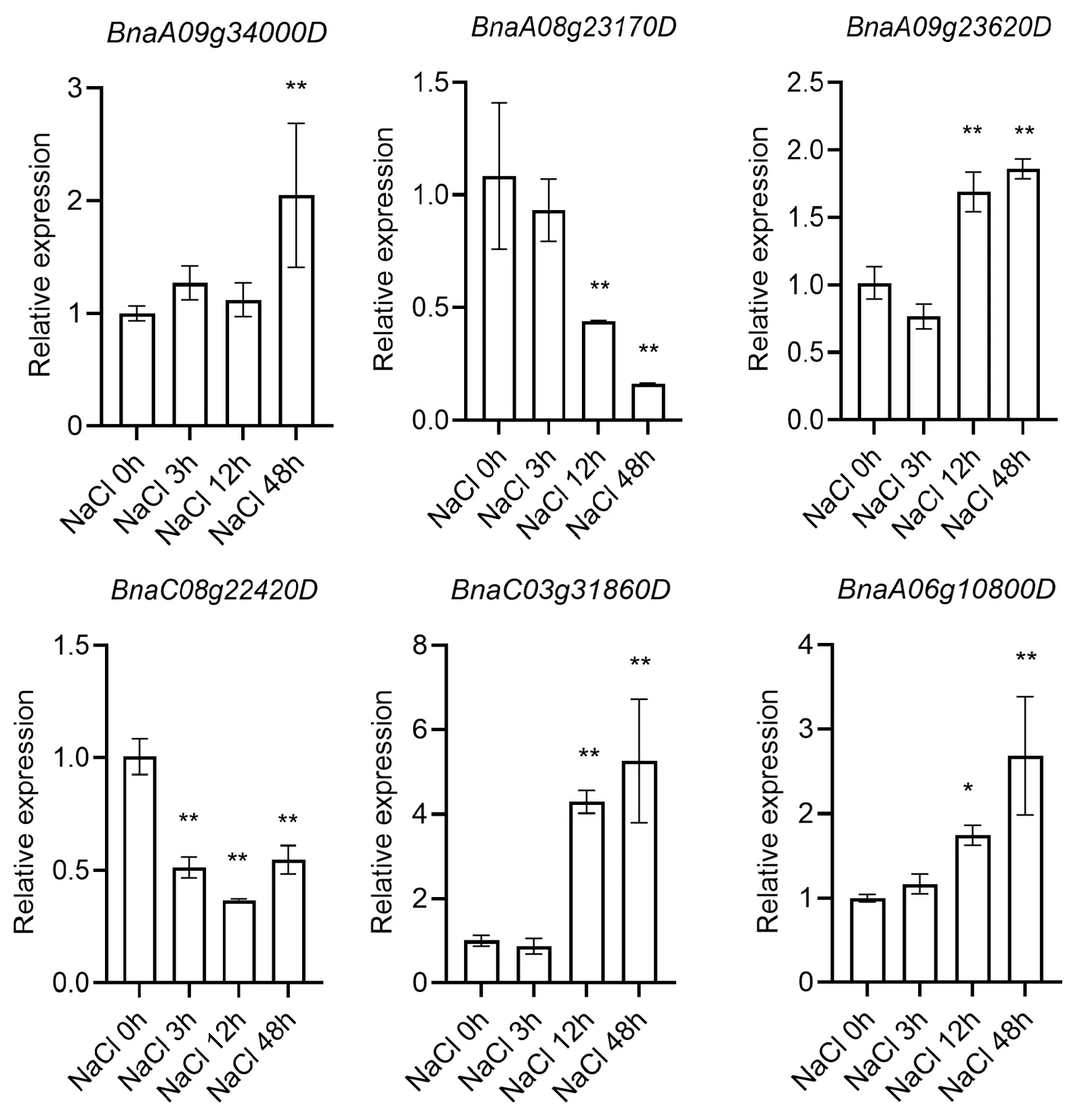
The relative expression analysis of the *BnaMEDs* under salt (NaCl) treatment. The x-axis represents different treatments. The y-axis shows relative expression levels normalized to the 0 h time point. Data are representative of three independent experiments (*n* = 3, mean *±* SD, * *p* < 0.05, ** *p* < 0.01, Student’s *t*-test).

The results revealed distinct time-specific and dose-dependent expression patterns of *BnaMED* genes under salt stress, which could be categorized into positive (up-regulated) and negative (down-regulated) responses to salt stress (Figure 7). Four *BnaMED* genes, including *BnaA09g34000D*, *BnaA09g23620D*, *BnaC03g31860D*, and *BnaA06g10800D*, were significantly up-regulated under salt stress, but with different temporal dynamics. Notably, *BnaC03g31860D* exhibited the most robust response, and *BnaA06g10800D* showed a time-dependent gradient up-regulation; *BnaA09g23620D* and *BnaA09g34000D* displayed delayed responses under treatment. On the other hand, two genes, *BnaA08g23170D* and *BnaC08g22420D*, were continuously and significantly down-regulated under salt stress. The activation of positive response genes and the suppression of negative response genes coordinate to facilitate the adaptation of *B. napus* to salt stress.

**Figure 8:**
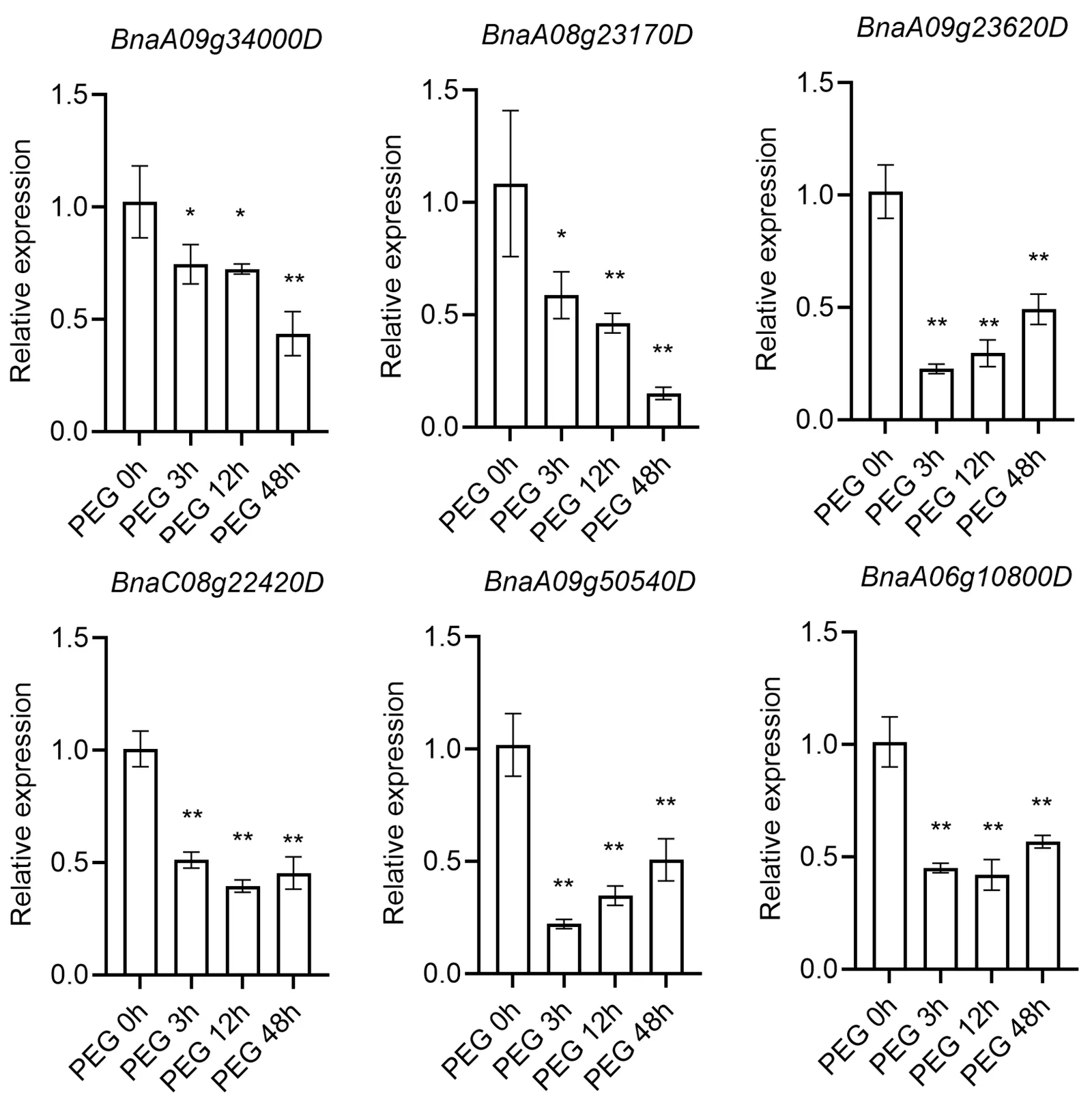
The relative expression analysis of the *BnaMEDs* under drought (PEG) treatment. The x-axis represents different treatments. The y-axis shows relative expression levels normalized to the 0 h time point. Data are representative of three independent experiments (*n* = 3, mean *±* SD, * *p* < 0.05, ** *p* < 0.01, Student’s *t*-test).

Under drought stress, six *BnaMED* genes also exhibited significant transcriptional responses. Unlike the diverse expression patterns detected under salt stress, all examined genes displayed a continuous downward trend in transcript abundance following drought treatment (Figure 8). This suggests that these family members may respond to drought stress in a relatively consistent manner in rapeseed. This universal down-regulation suggests that BnaMED genes likely act as negative regulators in the drought stress response. Alternatively, their functions may be actively repressed at the transcriptional level under water-deficit conditions to rewire cellular energy metabolism and orchestrate the growth–defense trade-off in plants. Notably, this expression pattern is in sharp contrast to the dynamic responses detected under salt stress, indicating that *BnaMED* genes may mediate abiotic stress tolerance through distinct regulatory strategies in response to different adverse environments.

In summary, the stress-specific expression profiles of the *BnaMED* gene family under drought and salt stress provide transcriptional evidence for their functional differentiation during stress adaptation in rapeseed, and also lay an important foundation for further dissecting their precise regulatory mechanisms within multiple stress-response networks.

### 2.9. Expression Profiling Analysis of BnaMED Genes During Floral Transition

Dynamic changes in gene expression during the transition from vegetative to reproductive growth are essential for understanding floral initiation. To investigate the expression dynamics of the *BnaMED* gene family throughout this developmental shift in *Brassica napus* cv. Zhong Shuang 11 (ZS11 or ZS), a semi-winter ecotype, we performed transcriptome analysis on three distinct meristem tissues including: shoot apical meristem (SAM) representing the pre-floral transition state, inflorescence meristem (IM) representing the ongoing floral transition state, and floral meristem (FM) representing the post-floral transition state. The expression levels of *BnaMED* genes across these developmental stages were quantified based on FPKM values and visualized using heatmap analysis based on our unpublished raw data.

**Figure 9:**
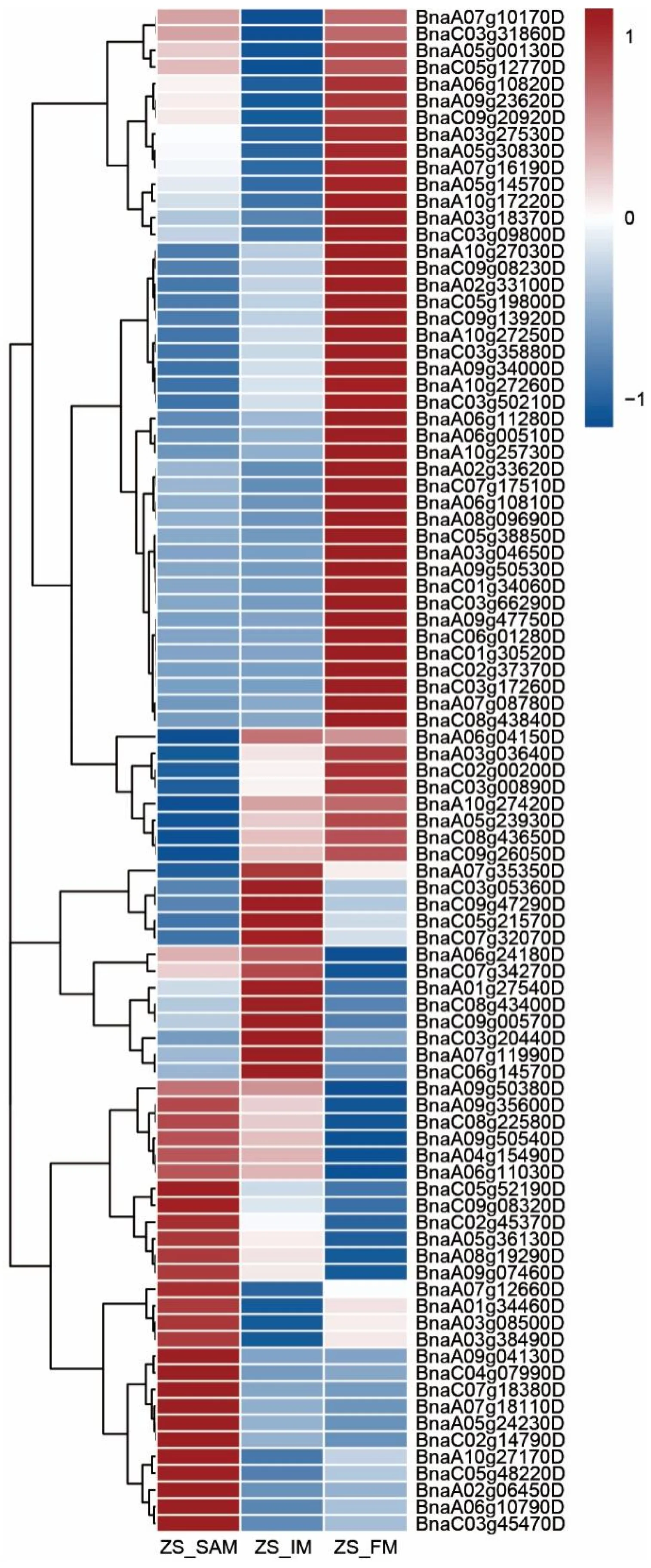
Expression profiles of *MED* family genes during floral transition in ZS11. SAM represents shoot apical meristem tissue, IM represents inflorescence meristem tissue, and FM represents floral meristem tissue. The heat map was generated based on FPKM values derived from transcriptome data. Color intensity indicates normalized gene expression levels, as shown in the scale bar on the right side of the figure. Red indicates relatively high expression levels, whereas blue indicates relatively low expression levels.

Heatmap analysis revealed that a total of 91 *BnaMED* genes were expressed during floral transition, and their expression patterns exhibited clear temporal specificity throughout reproductive development. Hierarchical clustering further classified these genes into four major groups based on their expression trends. Notably, a large group of 43 genes exhibited pronounced high expression specifically in the FM. These genes may play critical roles in floral organ initiation and maintenance. In contrast, 8 genes showed elevated expression levels in both the IM and FM, suggesting their potential involvement in multiple stages of reproductive growth. Additionally, 13 genes were found to be highly expressed exclusively in the IM, indicating that they may be vital for the initiation of floral transition. Conversely, 27 genes displayed high expression levels in the SAM, followed by a progressive decline in the IM and FM. This expression pattern suggests that these genes may act as negative regulators of reproductive growth, potentially maintaining the vegetative state prior to floral induction (Figure 9).

In summary, the *BnaMED* gene family exhibits dynamic and stage-specific expression patterns during the floral transition in *Brassica napus*. The identification of genes preferentially expressed in the SAM, IM, and FM provides valuable insights into their potential roles in repressing or promoting reproductive development. Furthermore, the functional divergence observed among different *BnaMED* genes during this critical phase highlights their complex regulatory landscape, which may have been shaped by polyploidization and subsequent neofunctionalization. These findings not only deepen our understanding of the involvement of the Mediator complex in floral transition but also provide a valuable set of candidate genes for future functional studies aimed at improving flowering time regulation and inflorescence architecture in rapeseed.

### 2.10. Functional Analysis of BnaMED Genes in Regulating Leaf Water Retention Under Drought and Salt Stress

Based on previous transcriptome screening of the *BnaMED* gene family, several members with differential expression in response to drought and salt stress were identified. To further clarify their biological functions in plant abiotic stress tolerance, three candidate genes including *BnaC08g22420D*, *BnaA09g23620D* and *BnaA09g34000D* were transiently over-expressed in *Nicotiana benthamiana* leaves. The detached leaves were treated with 20% PEG6000 and 200 mM NaCl to simulate drought and salt stress, respectively, and the leaf water loss rate was determined to evaluate the regulatory effects of these genes on plant water retention capacity under stress conditions.

**Figure 10:**
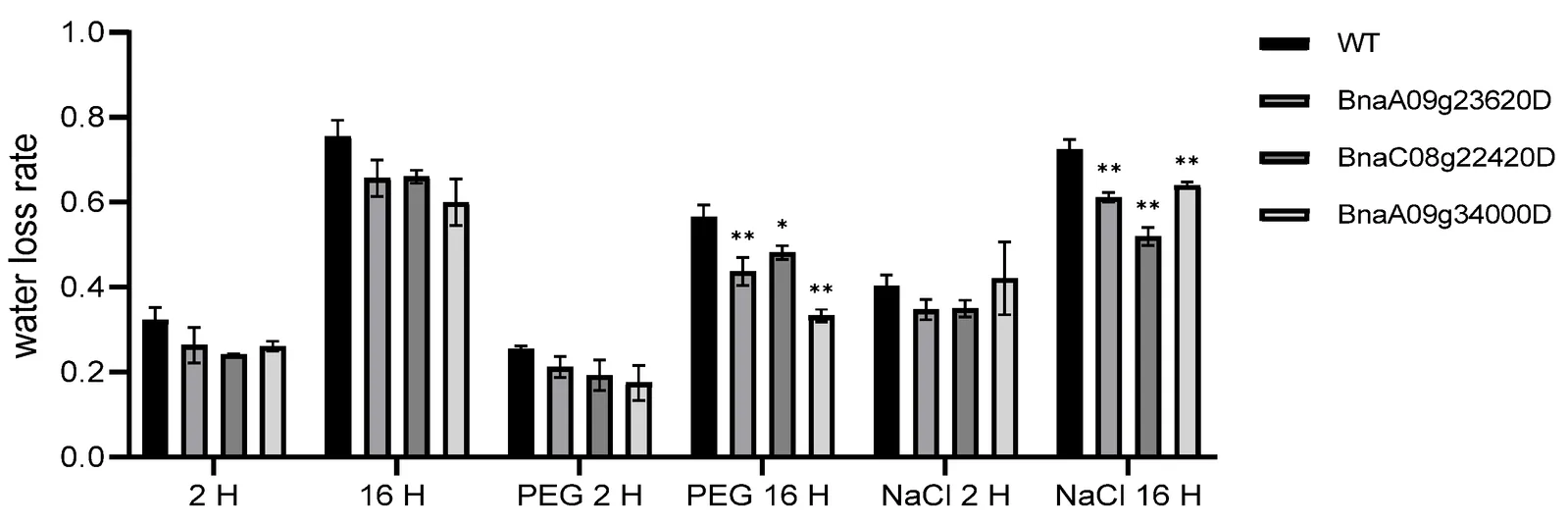
The water loss rates of detached *Nicotiana benthamiana* leaves transiently overexpressing *BnaA09g23620D*, *BnaA09g34000D*, and *BnaC08g22420D* were determined after 2 h and 16 h of dehydration, respectively. Leaves were pretreated with salt stress or drought stress. WT, wild-type tobacco blank control. Values represent mean ± SD (*n* = 3). * *p* < 0.05, ** *p* < 0.01.

Phenotypic analysis of leaf water loss showed that no significant differences in water loss rate were observed between transgenic leaves and wild-type controls after 2 h of drought or salt stress treatment. However, after 16 h of treatment, leaves overexpressing the three *MED* genes exhibited significantly lower water loss rates than wild-type leaves under both stress conditions (Figure 10). Based on the results presented in Section 2.8, the transcript level of endogenous *BnaC08g22420D* was significantly down-regulated under both drought and salt stress. By contrast, tobacco plants heterologously overexpressing *BnaC08g22420D* exhibited markedly lower leaf water loss rates compared with wild-type plants under drought and salt treatments. These findings indicate that *BnaC08g22420D* acts as a positive regulator of drought and salt stress tolerance in *Brassica napus*. Nevertheless, its endogenous transcription is strongly repressed upon stress exposure, suggesting that stress conditions suppress *BnaC08g22420D* at the transcriptional level, and reduced endogenous transcript abundance may constrain the stress-resistance capacity mediated by this gene. Heterologous overexpression can bypass such transcriptional repression and confer enhanced tolerance to abiotic stresses by elevating its protein abundance. In contrast, *BnaA09g23620D* and *BnaA09g34000D* exhibited stress-dependent differential endogenous expression: their transcripts were significantly up-regulated under salt stress yet repressed under drought stress. Nonetheless, heterologous overexpression of these two genes consistently alleviated leaf water loss and improved water-retention capacity under both stress treatments. This apparent discrepancy indicates that their endogenous transcript responses do not fully predict their overexpression-mediated physiological functions. Collectively, these data suggest that *BnaA09g23620D* and *BnaA09g34000D* act as positive regulators promoting plant stress tolerance.

## 3. Discussion

As the core hub of eukaryotic transcriptional regulation, the Mediator complex plays a key role in plant growth and development and stress adaptation. In this study, 189 BnaMED family members were systematically identified in *B. napus*, and their significant characteristics in genome distribution, evolutionary expansion, structural diversity and stress response regulation were revealed. These findings not only expand the understanding of the evolutionary dynamics of plant mediator complexes, but also provide a new case for the functional differentiation of gene families in polyploid crops.

Compared with *A. thaliana*, rice, soybean and other species, the number of *MED* genes (189) in *B. napus* was significantly increased, and there were a large number of collinearity homologous pairs between A/C subgenomes. Notably, the coexistence of “functional differentiation” and “redundancy buffering” is considered a key evolutionary strategy for polyploid crops to improve environmental adaptability and evolutionary potential. This directly confirms that heterologous polyploidization and subsequent genome-wide duplication events are the main driving forces for the expansion of the family. It is worth noting that soybean, as an ancient polyploid, also has a high number of *MED* genes and shows the most collinear pairs with rapeseed, which further supports the lineage-specific expansion pattern of the MED family in dicots. In contrast, the number of *MED* genes in monocots rice and maize is small, and the collinearity with rapeseed is extremely weak, indicating that the MED family may have experienced different retention and loss mechanisms after the differentiation of the two branches of angiosperms. This differentiation may be related to their unique transcriptional regulatory network needs—more complex developmental and metabolic pathways in dicots may require more mediator subunits to integrate diverse signals.

Phylogenetic analysis divided the *MED* genes into five subfamilies, of which the Unknown subfamily had the largest number of members, and 66 of the 79 were rapeseed proteins. The subfamily has fewer known members in *A. thaliana*, but is significantly amplified in rapeseed. We speculate that members of these unknown functions may have undergone subfunctionalization or neofunctionalization, and acquire species-specific regulatory roles after polyploidization. For example, they may be involved in the specific growth and development stages or environmental adaptability of rapeseed. In addition, these genes are highly heterogeneous in tissue expression profiles, for example, some are highly expressed in most tissues, while others are extremely lowly expressed. This suggests that functional differentiation may have occurred within the Unknown subfamily, which is worthy of further exploration through protein interaction and mutant phenotype analysis.

This study found that the vast majority of *BnaMED* genes contain three or more exons, providing a structural basis for alternative splicing. In plant stress response, alternative splicing is an important mechanism for rapid adjustment of protein function. Therefore, the multi-exon structure may give the *MED* gene the ability to produce multiple transcript subtypes when the environment changes. The existence of a few single-exon genes (such as *BnaC09g08320D*) is relatively rare. They are compact in structure and may be rapidly expressed by reducing the splicing link, which is suitable for performing certain transient or constitutive functions. This structural duality may reflect the family’s adaptation to different regulatory needs during evolution.

Promoter analysis showed that *BnaMED* genes were widely enriched in light response, hormone (ABA, MeJA, GA, etc.) and a variety of abiotic stress (low temperature, drought, anaerobic) response elements. Among them, the ABA response element (ABRE) appeared in 151 genes, the low temperature element (LTR) appeared in 97 genes, and the drought element (MBS) appeared in 92 genes. These data strongly support the function of the MED family as an environmental signal integration node from the transcriptional regulation level. It is worth noting that the presence of a large number of ABA and MeJA elements suggests that the family may be involved in the regulation of plant hormone-dependent gene expression reprogramming under salt, drought and other stresses. This is highly consistent with the conclusion that MED16 positively regulates salt stress tolerance through ABA signaling pathway, indicating that homologous genes in rapeseed may retain similar regulatory logic. However, some *BnaMED* genes were continuously down-regulated under drought stress, suggesting that the same family members may respond to different stresses through different expression directions, which further enriched the regulatory complexity of mediator complexes in stress signaling networks.

Based on the transcriptome analysis of 12 tissues, the *BnaMED* genes exhibited obvious tissue preference. In particular, the members of the Head and Middle subfamily, which are the core modules in *A. thaliana*, generally show a trend of low expression or even transcriptional silencing in rapeseed. This phenomenon may be due to the gene redundancy after polyploidization, which leads to the expression attenuation of some copies, or it may be that these genes are only induced at specific development stages or under stress conditions, while the transcriptome under normal growth conditions used in this study fails to capture its activity. In addition, almost all *BnaMED* genes have extremely low expression levels in pollen, which is consistent with the functional localization that the mediator complex is mainly involved in transcription initiation rather than meiosis or pollen-specific development. In general, the expression pattern of *MED* genes in *B. napus* showed a shift from ‘core module high expression’ to ‘unknown module broad spectrum expression’, which may reflect the rewiring of transcriptional regulatory network in polyploid rapeseed during long-term evolution.

The results of qRT-PCR showed that six *BnaMED* genes showed two dynamic patterns of up-regulation and down-regulation under salt stress, while all the detected genes were continuously down-regulated under drought stress. This stress-specific expression differentiation has important functional implications. Taking *A. thaliana MED16* as an example, it is known to be a positive regulator of salt stress, and its mutants are sensitive to osmotic stress. In this study, the *MED16* homologous gene *BnaA09g23620D* in rapeseed was significantly up-regulated under salt stress, which was highly conserved with the function of *A. thaliana*. However, it was down-regulated under drought stress, which contradicted the positive demand of this gene for osmotic stress in *A. thaliana*. One possible explanation is that although drought and salt stress share osmotic stress components, there are essential differences in ion toxicity, stomatal regulation and signal transduction time dynamics between them. As a polyploid, *B. napus* may evolve a more elaborate regulatory mechanism to actively inhibit the expression of *MED16* under drought conditions to reduce energy consumption or redistribute resources to other emergency pathways. In addition, it may also be due to the expression differentiation between different homologous copies after polyploidization. The *MED16* homologous gene detected in this study may be only one copy, while other undetected copies may respond to drought up-regulation. This hypothesis needs to be verified by genome-wide promoter activity analysis and CRISPR knockout.

Transcriptome profiling revealed that 91 of the 189 *BnaMED* genes were expressed during floral transition in *Brassica napus* cv. Zhongshuang 11 (Figure 9). This result suggests that some *BnaMED* members exhibit tissue- or stage-specific expression patterns, whereas others may have undergone functional redundancy or expression divergence following polyploidization. Most expressed genes showed elevated transcript levels in the inflorescence meristem (IM) and floral meristem (FM), indicating that the *BnaMED* family is broadly associated with reproductive development. In particular, genes highly expressed in the FM may participate in floral organ development. In contrast, several *BnaMED* genes displayed high expression in the shoot apical meristem (SAM), followed by reduced expression in the IM and FM, suggesting potential roles as negative regulators of flowering that help maintain vegetative growth prior to floral induction. As early-flowering rapeseed varieties are increasingly important for double- and multiple-cropping systems, these SAM-enriched *BnaMED* genes may represent promising targets for breeding. Targeted disruption of candidate flowering repressors through gene-editing approaches such as CRISPR/Cas9 could potentially accelerate floral transition and reproductive development. Further functional studies will be required to validate their regulatory roles and assess their breeding potential for developing early-flowering rapeseed cultivars.

This study is mainly based on bioinformatics analysis and transcriptome data and lacks direct biochemical and genetic evidence. For example, whether the proteins encoded by *BnaMED* genes are indeed assembled into functional Mediator complexes, and whether the unknown subfamily members physically interact with the known Head/Middle/Tail modules, remains to be verified by yeast two-hybrid or co-immunoprecipitation. In addition, although the prediction of promoter elements suggests multiple transcription factor binding sites, the specific upstream regulatory network is still unclear. In the future, we can combine ChIP-seq technology to draw its regulatory map. In terms of functional research, genes with significant differences in expression under stress, such as *BnaC03g31860D* up-regulated by salt stress and *BnaA09g23620D* continuously down-regulated by drought stress, should be preferentially selected. Phenotypic identification was performed using *A. thaliana* heterologous expression or rapeseed gene-edited mutants to clarify their specific contribution in stress resistance.

In this study, three *BnaMED* genes (*BnaC08g22420D*, *BnaA09g23620D*, and *BnaA09g34000D*) screened from transcriptome data were functionally characterized using a *Nicotiana benthamiana* transient overexpression system. All three genes positively regulate drought and salt tolerance by enhancing leaf water retention in a time-dependent manner, with no detectable phenotypic difference from wild type under short-term (2 h) stress but remarkably reduced leaf water loss under long-term (16 h) stress, indicating their primary roles in sustained abiotic stress adaptation. Notably, these genes exhibited distinct endogenous expression patterns under stress: *BnaC08g22420D* was transcriptionally repressed by both drought and salt stresses, while *BnaA09g23620D* and *BnaA09g34000D* were salt-induced but drought-repressed. Despite such variable native expression, heterologous overexpression of all three genes consistently improved stress tolerance. This expression-function discrepancy suggests that stress-induced transcriptional repression constrains the intrinsic stress resistance of rapeseed, and complex endogenous regulatory networks may alter native gene expression, whereas exogenous overexpression can bypass these limitations to exert stable positive functions in abiotic stress tolerance.

Limitations of this study should also be noted. Only BLASTp bidirectional homology search (E-value < 1 × 10^−5^) was used for MED family gene identification, without HMM-profile screening. Therefore, a small number of highly diverged MED members with low sequence conservation might be missed.

In summary, this study systematically described the evolutionary expansion, structural diversity, regulatory-network basis and stress-response characteristics of the *MED* gene family in *B. napus*, and revealed the profound influence of polyploidization events on the functional remodeling of the family. In particular, the distinct expression strategies under drought and salt stress provide a new perspective for understanding how polyploid crops integrate multiple stress signals through transcriptional regulatory networks. These findings lay an important theoretical foundation and candidate gene resources for subsequent utilization of key *MED* genes in genetic improvement of stress resistance in rapeseed.

## 4. Materials and Methods

### 4.1. Identification of the MED Family

Identification of the *MED* gene family was performed following the procedures below. First, The BLASTp program (e-value < 1 × 10^−5^) was employed to search for candidate MED protein sequences in the whole-genome protein dataset of *B. napus*, using AtMED family proteins as bait sequences. Subsequently, the candidate MED protein sequences of *B. napus* were screened, and only sequences containing the conserved MED motifs were retained as members of the *MED* gene family.(For example, AtMED11 and AtMED12 genes contain MED11 conserved domain and MED12 conserved domain, respectively. We found sequences containing MED11 domain or MED12 domain as homologous genes of AtMED11 and AtMED12 genes.) Genes inconsistent with the structural and functional characteristics of the MED family were excluded. All analytical results were visualized using TBtools-II v2.382 software [41]. The protein sequences used in this study were retrieved from the following public databases. The *A. thaliana* Information Resource (TAIR10, https://www.arabidopsis.org/ (accessed on 1 September 2025)), the *B. napus* Multi-omics Information Resource (BnIR, https://yanglab.hzau.edu.cn/ (accessed on 16 September 2025)), and the National Center for Biotechnology Information (NCBI, https://www.ncbi.nlm.nih.gov (accessed on 1 October 2025)).

### 4.2. Sequence Alignment and Phylogeny Analysis of BnaMEDs

Multiple sequence alignment and phylogenetic analysis were performed on the *BnaMED* gene family to explain its evolutionary relationships. First, protein sequences of *MED* genes from *B. napus* and *A. thaliana* were aligned using the MUSCLE algorithm implemented in MEGA X 10.2.6 (64-bit, https://www.megasoftware.net/). A phylogenetic tree was then constructed using the Neighbor-Joining (NJ) method with 1000 bootstrap replicates. Finally, the phylogenetic tree was visualized and annotated using the online tool iTOL (https://itol.embl.de/ (accessed on 10 October 2025)). Under normal circumstances, the grouping of homologous genes is grouped by similar structures or motifs, but because the BnaMED family is relatively special, because its homology is not very high, if the homology grouping will be divided into many subunits, so we retreat to the next, according to the five parts of *Arabidopsis thaliana* to group.

### 4.3. Chromosome Location

To determine the chromosomal distribution of *BnaMED* genes in *B. napus*, a chromosome-location map of the *BnaMED* gene family was constructed using TBtools v2.310 software. The genome annotation file Westar.v0.GFF3 used in this analysis was retrieved from the National Center for Biotechnology Information (NCBI) (https://www.ncbi.nlm.nih.gov/ (accessed on 25 October 2025)).

### 4.4. Synteny Analysis of BnaMED Genes

To investigate the collinearity of *BnaMED* genes with those in other plant species, we used the One-Step McScanX tool implemented in TBtools v2.310 to generate collinearity files. Genome data for *B. napus* (Da-Ae), *A. thaliana* (TAIR 10.1), *G. max* (Glycine_max_v4.0), *O. sativa* L. (AGIS 1.0), and *Z. mays* (Zm-B73-REFERENCE-NAM- 5.0) were obtained from the National Center for Biotechnology Information (NCBI, https://www.ncbi.nlm.nih.gov (accessed on 29 October 2025)). Collinearity results were visualized using TBtools v2.310.

### 4.5. The Biophysical Properties of BnaMEDs

The physicochemical properties of BnaMED proteins, including molecular weight (Mw), amino-acids count (AA), and theoretical isoelectric point (pI), were analyzed using TBtools-II v2.382 software.

### 4.6. The Identification of Cis-Regulatory Elements in Promoter Regions

To identify putative cis-acting regulatory elements in *BnaMED* genes, the 2.0 kb genomic sequence upstream of the ATG initiation codon of each gene was analyzed using PlantCARE (https://bioinformatics.psb.ugent.be/webtools/plantcare/html/ (accessed on 2 November 2025)). The results were visualized using TBtools-II v2.382.

### 4.7. Expression of BnaMED in Different Tissues

The expression data of *BnaMED* genes across different tissues were obtained from the BnIR database (https://yanglab.hzau.edu.cn/ (accessed on 5 November 2025)), and the corresponding heat-map was generated with TBtools-II v2.382.

### 4.8. Expression Analysis of BnaMED Genes During Floral Transition

Expression data of *BnaMED* genes in shoot apical meristem (SAM), inflorescence meristem (IM), and floral meristem (FM) tissues during floral transition in ZS11 were obtained from unpublished in-house transcriptome datasets (Figure 9). Heatmaps illustrating gene expression patterns were generated using the R package pheatmap.

### 4.9. RNA Isolation and Quantitative Real-Time PCR (qRT-PCR) Analysis

In the gene-expression analysis, 7-day-old wild-type Westar seedlings were placed in a black plastic culture container and cultured with Hoagland nutrient solution. After 14 days, the roots of seedlings were exposed to simulated drought stress with 20% PEG-6000 and salt stress with 200 mM sodium chloride solution. Four treatment time points were set, including 0 h (control group), 3 h, 12 h and 48 h. Leaf tissue samples were collected immediately after each treatment for subsequent RNA extraction. Total RNA was extracted using the Plant (polysaccharides and polyphenols) RNA extraction kit (Easy-DO, Hangzhou, China), and then its quality was verified and its concentration was determined. FastKing gDNA Dispelling RT SuperMix (Beijing, China) was used for cDNA synthesis, and UltraSYBR mixture (Cwbio, Taizhou, China) combined with CFX Opus 96 real-time fluorescence quantitative PCR system (BIO-RAD, Hercules, CA, USA) was used for qRT-PCR detection. The gene of *BnaActin* served as a reference gene. Three biological replicates were included in the expression analysis. Primer sequence details are shown in Table S1. Each sample was analyzed with three biological and three technical replicates to guarantee experimental reliability. Relative gene expression levels were quantified via the standardized 2 method. Briefly, ΔCt was calculated as the Ct value of the target gene minus that of the internal reference gene.

### 4.10. Stress Treatment, Water Loss Rate Measurement in Transiently Transformed Tobacco Leaves

A 1300-35S overexpression vector with a 3FLAG tag was constructed to drive the ectopic expression of rapeseed *BnaC08g22420*, *BnaA09g23620D*, and *BnaA09g34000D* genes under the control of the CaMV 35S promoter. The recombinant vector was transiently infiltrated into *Nicotiana benthamiana* leaves via the GV3101-mediated method. Infiltrated tobacco plants were dark-cultured at 25 ± 1 °C for 24 h for recovery, followed by 24 h of continuous light. Uniform and healthy tobacco leaves were subjected to 24 h of salt stress (200 mM NaCl) and simulated drought stress (20% PEG-6000) at 25 °C, with untreated wild-type tobacco leaves serving as controls. All groups were set with three biological replicates to ensure experimental reliability. After treatment, fresh leaf samples were collected for water loss measurement. Leaf water loss rate was measured with minor modifications to standard protocols. Treated leaves were rinsed with deionized water and blotted dry gently, followed by immediate weighing to record the initial fresh weight (FW_0_). Leaves were placed at 25 °C under ventilated dark conditions for natural dehydration, and their fresh weights were re-measured at 2 h and 16 h. The water loss rate was calculated as follows: Water loss rate (%) = (Initial fresh weight − Dehydrated fresh weight)/Initial fresh weight × 100%.

## 5. Conclusions

This study performed comprehensive genome-wide identification and systematic characterization of the *MED* gene family in *Brassica napus*, and a total of 189 *BnaMED* genes were identified and analyzed from the perspectives of genomic distribution, evolutionary collinearity, phylogenetic classification, gene structure, cis-regulatory elements, and spatiotemporal and stress-induced expression patterns. Our results revealed the uneven chromosomal distribution and lineage-specific expansion of *BnaMED* genes, as well as conserved evolutionary collinearity with dicotyledonous species and weak synteny with monocotyledonous plants; phylogenetic analysis further classified the BnaMED family into five subfamilies with obvious structural heterogeneity and functional differentiation, and abundant light-, hormone-, and abiotic stress-responsive cis-elements were detected in their promoters, indicating their essential roles in environmental signal integration and developmental regulation. Transcriptome data combined with qRT-PCR validation confirmed that *BnaMED* genes exhibit distinct expression profiles under salt and drought stress, with specific members displaying divergent regulatory functions in abiotic stress adaptation, and key family members show preferential expression in shoot and inflorescence meristems, implying their potential roles in rapeseed growth and development. Functional verification further demonstrated that three core *BnaMED* genes exert differentiated regulatory effects on drought and salt stress tolerance by modulating leaf water retention and water homeostasis, underlying the sophisticated molecular regulatory network of rapeseed in response to abiotic stresses. Collectively, this work systematically clarifies the evolutionary characteristics, expression patterns, and stress-related biological functions of the *BnaMED* gene family, provides reliable molecular evidence and valuable candidate gene resources for dissecting MED-mediated stress tolerance mechanisms in *B. napus*, and lays a solid theoretical foundation for the genetic improvement and molecular breeding of stress-resistant rapeseed varieties.

## Figures and Tables

**Table 1:** Information regarding the *BnaMED*.

Sequence ID	Number of Amino Acid	Molecular Weight	Theoretical pI	Instability Index	Aliphatic Index	Grand Average of Hydropathicity
BnaUnn_Randomg17000D	255	28,429.11	5.02	58.81	74.98	−0.475
BnaUnn_Randomg12830D	1807	202,232.79	8.92	56.53	62.47	−0.717
BnaUnn_Randomg04920D	1912	207,974.47	5.63	53.6	77.15	−0.267
BnaUnn_Randomg05040D	257	29,055.12	6.78	58.01	88.68	−0.472
BnaUnn_Randomg68910D	650	71,421.68	5.24	36.58	82.34	−0.442
BnaUnn_Randomg29650D	848	96,058.08	7.5	47.84	85.18	−0.3
BnaUnn_Randomg73580D	411	46,708.26	6.59	52.45	67.18	−0.904
BnaUnn_Randomg07270D	1002	114,834.19	6.68	48.93	58.7	−1.102
BnaUnn_Randomg13810D	827	91,929	7.79	49.49	63.86	−0.856
BnaUnn_Randomg17440D	255	28,402.09	5.02	58.81	74.98	−0.465
BnaUnn_Randomg07360D	1626	181,383.57	8.92	55.21	62.65	−0.693
BnaUnn_Randomg72390D	1610	179,662.72	8.86	54.46	63.27	−0.667
BnaUnn_Randomg36800D	314	35,878.84	4.76	52.81	75.7	−0.889
BnaC09g00570D	2165	239,795.18	8.87	45.19	89.55	−0.266
BnaC09g03650D	1272	142,796.3	9.24	65.97	75.73	−0.657
BnaC09g08230D	162	17,124.88	4.64	44.09	78.4	−0.415
BnaC09g08320D	468	52,614.13	9.23	41.06	78.78	−0.477
BnaC09g13920D	190	21,865.19	9.2	41.59	78.58	−0.634
BnaC09g20920D	467	52,925	9.02	45.47	108.05	0.129
BnaC09g26050D	77	8522.72	5.14	36.74	86.1	−0.165
BnaC09g26060D	413	45,961.47	9.74	64.53	54.87	−0.933
BnaC09g27600D	1250	135,005.77	5.68	45.16	75.63	−0.219
BnaC09g27770D	111	11,543.06	4.76	57.38	77.84	−0.243
BnaC09g46770D	825	93,639.55	9.11	59.51	75.47	−0.586
BnaC09g47290D	204	23,736.48	9.52	39.17	50.25	−1.677
BnaC09g53180D	234	27,219.65	9.41	42.91	52.09	−1.512
BnaC09g55320D	341	38,994.12	5.21	63.11	61.2	−1.141
BnaC09g58310D	435	48,863.51	5.83	50.59	66.41	−0.909
BnaC09g59810D	160	18,675.28	6.98	68.3	96.25	−0.633
BnaC09g60210D	665	72,982.92	5.05	35.35	85.53	−0.346
BnaC08g11920D	707	79,454.84	7.22	44.18	86.29	−0.381
BnaC08g20520D	345	39,223.55	6.43	47.22	84.43	−0.484
BnaC08g22420D	1461	160,353.55	9.72	61.42	68.52	−0.594
BnaC08g22580D	1806	200,468.99	8.67	54.62	65.41	−0.664
BnaC08g34840D	432	48,595.57	6.89	41.4	95.19	−0.126
BnaC08g43400D	2007	223,822.62	8.8	48.44	68.64	−0.636
BnaC08g43650D	144	16,344.36	4.68	43.29	82.57	−0.678
BnaC08g43840D	1039	114,010.22	9.27	69.12	72.68	−0.658
BnaC08g47700D	650	72,363.09	4.91	32.06	85.92	−0.468
BnaC07g11570D	668	73,673.38	5.11	29.07	84.96	−0.473
BnaC07g12980D	1309	143,216.53	6.88	46.09	102.8	0.144
BnaC07g15030D	275	32,199.95	6.07	67.33	69.85	−1.171
BnaC07g17510D	668	73,535.8	6.57	35.78	90.9	−0.345
BnaC07g18380D	1554	173,750.14	8.19	44.75	96.6	−0.053
BnaC07g28370D	259	29,005.04	5.06	61.86	84.02	−0.532
BnaC07g32070D	1044	117,577.8	9.06	69.31	63.25	−0.895
BnaC07g34270D	1033	116,401.02	8.8	48.15	80.45	−0.411
BnaC07g40120D	903	100,671.36	5.85	42.54	84.41	−0.473
BnaC06g01280D	937	108,035.46	6.09	55.72	61.21	−0.998
BnaC06g02940D	354	40,249.43	5.81	39.45	70.28	−0.888
BnaC06g03080D	503	56,046.56	5.28	38.29	80.66	−0.496
BnaC06g09160D	207	24,071.1	9.19	50	91.4	−0.451
BnaC06g09570D	1893	204,467.4	5.81	45.83	77.98	−0.232
BnaC06g14440D	211	23,722.01	6.9	41.3	86.35	−0.457
BnaC06g14570D	1937	211,142.27	5.55	53.04	78.31	−0.265
BnaC06g18060D	612	67,682.41	5	31.17	83.95	−0.534
BnaC06g18330D	88	10,076.61	9.26	62.73	89.77	−0.685
BnaC06g43290D	1610	179,598.35	8.81	54.64	62.28	−0.709
BnaC05g12570D	2787	311,668.83	9.08	62.01	75.41	−0.66
BnaC05g12770D	647	71,045.47	5.43	35.66	83.34	−0.423
BnaC05g13100D	667	74,173.51	8.86	36.27	79.27	−0.384
BnaC05g19800D	1502	167,939.94	6.85	43.72	97.2	−0.064
BnaC05g21570D	701	79,219.41	6.11	39	87.39	−0.476
BnaC05g36310D	272	30,265.31	5.21	56.74	78.16	−0.378
BnaC05g38850D	826	91,977.18	8.05	51.19	63.21	−0.857
BnaC05g39170D	1012	115,905.77	6.72	48.09	60.51	−1.015
BnaC05g48220D	420	47,890.73	6.6	49.87	66.19	−0.962
BnaC05g51360D	601	67,679.04	5.43	42.77	79.65	−0.4
BnaC05g52190D	1629	177,075.24	7.06	45.63	95.28	−0.092
BnaC05g54040D	115	13,201.03	6.1	56.53	87.22	−0.497
BnaC04g00060D	1701	187,688.5	6.81	44.53	99.12	0.004
BnaC04g00230D	338	38,393.61	6.26	34.82	124.32	0.094
BnaC04g00240D	1047	113,330.63	6.4	43.7	100.71	0.197
BnaC04g07990D	104	12,233.78	5.22	40.4	62.02	−1.273
BnaC04g13320D	229	25,736.65	5.85	47.76	101.22	0.059
BnaC04g19760D	220	25,185.96	7.82	35.07	88.55	−0.217
BnaC04g41810D	218	23,340.1	6.23	40.25	105.92	0.297
BnaC04g48330D	473	51,863.76	5.04	35.38	82.96	−0.481
BnaC03g00890D	511	56,792.92	6.55	44.83	71	−0.617
BnaC03g02110D	390	43,792.33	7.63	45.25	69.28	−0.821
BnaC03g04120D	350	40,009.58	5	56.82	77.4	−0.87
BnaC03g05360D	221	25,765.75	9.52	39.11	46.79	−1.672
BnaC03g09800D	644	72,084.58	5.84	45.95	87.17	−0.38
BnaC03g17260D	831	92,138.79	5.6	34.65	84.6	−0.513
BnaC03g20440D	669	73,641.31	5.08	28.21	85.41	−0.464
BnaC03g31860D	2222	246,415.88	8.8	48.24	89.32	−0.274
BnaC03g35880D	429	49,191.36	9.14	44.27	86.11	−0.662
BnaC03g37190D	1622	183,367.72	8.74	51.56	64.66	−0.688
BnaC03g45470D	533	60,151.62	5.43	59.73	78.76	−0.177
BnaC03g46040D	362	40,192.65	4.83	36.57	87.02	−0.343
BnaC03g46800D	183	21,028.2	6.83	42.72	85.79	−0.427
BnaC03g50210D	176	18,415.27	4.61	40.76	78.86	−0.397
BnaC03g66290D	707	79,255.43	8.99	52.43	83.55	−0.47
BnaC03g76010D	241	28,076.85	6.01	80.33	55.35	−1.199
BnaC02g00200D	394	44,429.78	5.85	46	72.34	−0.822
BnaC02g00850D	814	91,898.26	7.23	47.85	85.49	−0.288
BnaC02g07410D	813	92,533.81	9.18	59.38	70.91	−0.652
BnaC02g14790D	131	14,403.25	5.09	59.14	93.05	−0.461
BnaC02g28910D	1749	194,756.51	8.56	56.53	66.94	−0.629
BnaC02g37370D	536	58,972.88	5.06	33.37	88.23	−0.304
BnaC02g45370D	252	29,678.49	6.21	42.06	100.2	−0.123
BnaC01g30520D	599	66,416.58	5.27	35.95	86.29	−0.387
BnaC01g34060D	281	30,655.71	9.18	36.85	104.09	0.132
BnaC01g43590D	1717	193,767.1	8.84	51.66	69.59	−0.592
BnaC01g43910D	474	52,217.15	5	33.17	83.59	−0.48
BnaC01g46170D	508	56,420.21	5.06	36.7	86.24	−0.419
BnaA10g06650D	943	108,222.89	5.82	58.87	54.41	−1.134
BnaA10g17220D	821	93,357.07	9	56.73	77.25	−0.585
BnaA10g17490D	236	27,117.64	9.52	43.53	62.42	−1.261
BnaA10g21610D	243	28,258.77	9.49	34.05	50.16	−1.481
BnaA10g23140D	338	38,794.04	5.39	66.99	63.43	−1.123
BnaA10g25730D	452	51,056.21	6.28	52.6	66.92	−0.901
BnaA10g27030D	184	21,228.1	5.57	80.95	87.39	−0.582
BnaA10g27170D	792	87,056.54	5.55	63.74	78.98	−0.456
BnaA10g27250D	701	77,300.14	5.43	36.66	85.46	−0.356
BnaA10g27260D	696	76,610.32	5.33	35.79	85.66	−0.346
BnaA10g27420D	792	87,056.54	5.55	63.74	78.98	−0.456
BnaA09g00900D	2256	249,732.32	8.91	46.21	89.01	−0.252
BnaA09g04130D	1478	166,400.18	8.6	63.29	72.83	−0.7
BnaA09g07460D	517	56,699.14	4.95	35.11	83.62	−0.442
BnaA09g08160D	167	17,885.09	4.85	37.63	84.79	−0.281
BnaA09g08240D	468	52,640.17	9.23	42.82	78.57	−0.485
BnaA09g22390D	435	48,348.55	7.86	54.57	67.75	−0.728
BnaA09g23620D	1325	143,956.03	5.73	45.8	75.48	−0.257
BnaA09g34000D	769	82,584.31	8.99	56.65	74.84	−0.33
BnaA09g34380D	870	96,441.44	5.72	37.64	89.41	−0.466
BnaA09g35600D	1518	169,944.15	7.26	43.54	96.57	−0.07
BnaA09g47750D	150	16,796.88	7.13	33.99	111.07	0.307
BnaA09g50150D	1971	219,034.12	8.48	49.82	67.68	−0.661
BnaA09g50380D	226	25,219.02	8.17	47.19	84.51	−0.18
BnaA09g50530D	141	15,763.93	5.61	36.84	82.34	−0.467
BnaA09g50540D	1260	138,536.89	9.42	72.71	68.28	−0.761
BnaA09g53770D	553	61,566.92	4.96	31.93	85.82	−0.505
BnaA08g00980D	237	27,548.33	6.01	75.6	56.71	−1.149
BnaA08g09690D	704	79,154.49	7.52	42.62	86.09	−0.385
BnaA08g19290D	738	82,745.56	8.83	51.18	83.88	−0.444
BnaA08g23170D	835	89,232.37	8.93	60.55	69.63	−0.437
BnaA08g26300D	1186	134,781.27	8.34	53.18	68.41	−0.666
BnaA08g26470D	1455	157,962.98	9.19	54.39	68.25	−0.52
BnaA07g07840D	669	73,848.58	5.08	30	84.68	−0.477
BnaA07g08780D	1314	143,693.93	6.43	46.1	103.36	0.157
BnaA07g10170D	274	32,378.07	5.74	67.01	65.84	−1.215
BnaA07g11990D	615	67,119.36	5.88	41.21	95.28	−0.223
BnaA07g12660D	1545	173,125.29	7.29	45.5	96.65	−0.042
BnaA07g16190D	254	28,809.32	8.87	39.03	90.91	−0.157
BnaA07g17980D	665	73,509.22	5.11	31.28	84.9	−0.47
BnaA07g18110D	91	10,619.28	8.76	50.31	85.82	−0.544
BnaA07g35350D	1604	179,346.22	8.81	54.28	62.58	−0.708
BnaA06g00510D	1883	203,357.07	5.69	45.98	77.91	−0.236
BnaA06g04150D	433	47,879.44	5.61	33.1	85.38	−0.41
BnaA06g10790D	1343	148,098.98	9.47	68.28	65.57	−0.719
BnaA06g10800D	759	85,088.65	9.84	66.11	71.05	−0.786
BnaA06g10810D	656	75,558.59	9.54	59.37	77.84	−0.781
BnaA06g10820D	965	107,881.22	8.38	43.16	84.32	−0.477
BnaA06g11030D	647	71,044.48	5.38	35.55	83.79	−0.407
BnaA06g11280D	646	71,464.34	8.59	35.54	79.71	−0.391
BnaA06g24180D	175	18,321.11	4.48	45.16	78.74	−0.413
BnaA06g31900D	1063	119,429.85	9.15	71.68	65.63	−0.891
BnaA06g34520D	267	30,041.09	5.25	55.27	81.46	−0.606
BnaA05g00130D	1319	143,526.56	5.96	42.74	104.79	0.195
BnaA05g14440D	1919	208,293.88	5.57	52.82	77.38	−0.261
BnaA05g14570D	427	49,156.47	9.22	57.85	88.29	−0.543
BnaA05g23930D	829	92,317.47	8.08	50.35	64.52	−0.856
BnaA05g24230D	967	110,807.11	6.27	46.42	62.52	−1.015
BnaA05g30830D	385	43,936.08	5.72	52.24	72.44	−0.944
BnaA05g33770D	627	70,730.38	5.47	46.07	77.59	−0.424
BnaA05g34860D	1645	178,851.28	7.08	46	93.29	−0.108
BnaA05g36130D	115	13,338.21	5.84	48.94	86.35	−0.565
BnaA04g15490D	218	23,376.17	6.23	39.02	102.34	0.279
BnaA03g01870D	403	45,546.98	5.73	47.74	66.55	−0.905
BnaA03g03640D	342	38,828.83	5.07	66.66	62.4	−1.094
BnaA03g04650D	249	28,849.38	9.39	36.26	62.97	−1.273
BnaA03g08500D	645	72,134.59	5.7	44.77	86.59	−0.379
BnaA03g15270D	669	73,631.28	5.08	27.79	84.83	−0.468
BnaA03g18370D	669	73,579.24	5.08	27.31	85.84	−0.461
BnaA03g27530D	2221	246,117.33	8.88	49.53	88.1	−0.283
BnaA03g31250D	480	55,291.12	6.22	38.11	87.31	−0.522
BnaA03g32060D	1581	178,628.66	8.73	52.38	66.34	−0.664
BnaA03g38160D	200	22,646.88	5.61	50.91	75.05	−0.589
BnaA03g38490D	472	52,270.24	5.16	33.07	83.5	−0.478
BnaA02g06450D	775	88,459.62	9.43	58.37	75.66	−0.605
BnaA02g23250D	147	16,443.78	9.73	62.67	79.66	−0.658
BnaA02g33100D	880	99,843.87	6.2	65.29	69.78	−0.78
BnaA02g33620D	252	29,708.53	6.15	40.36	98.25	−0.127
BnaA01g18700D	883	98,357.31	5.71	44.12	83.9	−0.491
BnaA01g24580D	441	48,958.52	5.23	34.49	84.49	−0.484
BnaA01g27540D	636	70,131.31	7.47	39.96	98.79	0.139
BnaA01g34460D	1783	200,736.13	8.85	50.11	66.43	−0.677

## Data Availability

The data presented in this study are available in the paper and Supplementary Files.

## Supplementary Materials

The following supporting information can be downloaded at https://www.mdpi.com/article/10.3390/plants15182877/s1: Table S1: Primers used for RT-qPCR; Table S2: Number of hormone and stress-responsive cis-elements in the promoters of the BnaMEDs.
