# Supplementary material for: Integrated Phylogenomics and Expression Profiling of the MED Gene Family in Brassica napus Uncover Their Roles in Plant Development and Stress Tolerance
Source: Plants (Basel). 2026 Sep 20;15(18):2877. doi: 10.3390/plants15182877 (PMC13610452; doi:10.3390/plants15182877)
Supplement: Supplementary file 1 [file plants-15-02877-s001.zip › Supplemeatary Table S2.pdf]

Supplementary Table S2. Number of hormone and stress-responsive cis-elements in the promoters of the BnaMEDs

Cis-Elements	Function of Cis-Elements	Number of Genes
3-AF1 binding site	light responsive element	17
4cl-CMA1b	light responsive element	1
A-box	cis-acting regulatory element	41
AAAC-motif	light responsive element	6
AACA_motif	involved in endosperm-specific negative expression	4
ABRE	cis-acting element involved in the abscisic acid responsiveness	151
ACA-motif	part of gapA in (gapA-CMA1) involved with light responsiveness	3
ACE	cis-acting element involved in light responsiveness	41
AE-box	part of a module for light response	82
ARE	cis-acting regulatory element essential for the anaerobic induction	180
AT-rich element	binding site of AT-rich DNA binding protein (ATBP-1)	36
AT-rich sequence	element for maximal elicitor-mediated activation (2copies)	18
AT1-motif	part of a light responsive module	13
ATC-motif	part of a conserved DNA module involved in light responsiveness	16
ATCT-motif	part of a conserved DNA module involved in light responsiveness	41
AuxRE	part of an auxin-responsive element	2
AuxRR-core	cis-acting regulatory element involved in auxin responsiveness	20
Box 4	part of a conserved DNA module involved in light responsiveness	158
Box II	part of a light responsive element	14
Box II -like sequence	cis-acting regulatory element	1
Box III	protein binding site	13
C-box	cis-acting regulatory element involved in light responsiveness	1
CAAT-box	common cis-acting element in promoter and enhancer regions	188
CAG-motif	part of a light response element	8
CCAAT-box	MYBHv1 binding site	58
CGTCA-motif	cis-acting regulatory element involved in the MeJA-responsiveness	131
chs-CMA1a	part of a light responsive element	30
chs-CMA2a	part of a light responsive element	17
chs-CMA2b	part of a light responsive element	2
chs-CMA2c	part of a light responsive element	2
chs-Unit 1 m1	part of a light responsive element	5
circadian	cis-acting regulatory element involved in circadian control	44
G-box	cis-acting regulatory element involved in light responsiveness	157
GA-motif	part of a light responsive element	37
Gap-box	part of a light responsive element	11
GARE-motif	gibberellin-responsive element	45
GATA-motif	part of a light responsive element	74
GATT-motif	part of a light responsive element	7
GC-motif	enhancer-like element involved in anoxic specific inducibility	19
GCN4_motif	cis-regulatory element involved in endosperm expression	30
GGA-motif	part of a light responsive element	1
GT1-motif	light responsive element	138
GTGGC-motif	part of a light responsive element	10
HD-Zip 1	element involved in differentiation of the palisade mesophyll cells	11
HD-Zip 3	protein binding site	12
I-box	part of a light responsive element	58
L-box	part of a light responsive element	2
LAMP-element	part of a light responsive element	26
LTR	cis-acting element involved in low-temperature responsiveness	97
MBS	MYB binding site involved in drought-inducibility	92
MBSI	MYB binding site involved in flavonoid biosynthetic genes regulation	15
MRE	MYB binding site involved in light responsiveness	67
MSA-like	cis-acting element involved in cell cycle regulation	18
NON-box	cis-acting regulatory element related to meristem specific activation	1

O2-site	cis-acting regulatory element involved in zein metabolism regulation	65
P-box	gibberellin-responsive element	55
Pc-CMA2a	part of a light responsive element	3
RY-element	cis-acting regulatory element involved in seed-specific regulation	6
SARE	cis-acting element involved in salicylic acid responsiveness	6
Sp1	light responsive element	29
TATC-box	cis-acting element involved in gibberellin-responsiveness	27
TC-rich repeats	cis-acting element involved in defense and stress responsiveness	81
TCA-element	cis-acting element involved in salicylic acid responsiveness	57
TCCC-motif	part of a light responsive element	44
TCT-motif	part of a light responsive element	115
TGA-box	part of an auxin-responsive element	6
TGA-element	auxin-responsive element	83
TGACG-motif	cis-acting regulatory element involved in the MeJA-responsiveness	131
WUN-motif	wound-responsive element	4
